# Acanthamoeba Keratitis Management and Prognostic Factors: A Systematic Review

**DOI:** 10.3390/jcm14072528

**Published:** 2025-04-07

**Authors:** Pedro Marques-Couto, Mariana Monteiro, Ana Margarida Ferreira, João Pinheiro-Costa, Rodrigo Vilares-Morgado

**Affiliations:** 1 Department of Ophthalmology, Unidade Local de Saúde São João, 4200-319 Porto, Portugal; 2 Faculty of Medicine, University of Porto, 4200-319 Porto, Portugal; 3 RISE-Health, Surgery and Physiology Department, Faculty of Medicine, University of Porto, 4200-319 Porto, Portugal

**Keywords:** Acanthamoeba, Acanthamoeba Keratitis, corneal infection, amebicides, keratoplasty, therapeutic epithelial debridement, prognostic factors

## Abstract

**Background/Objectives**: The aim of this study was to review the therapeutic and prognostic factors influencing Acanthamoeba Keratitis (AK) management. **Methods**: A systematic search was performed across MEDLINE^®^ (via PubMed), Web of Science^®^, and Scopus^®^, following the PRISMA 2020 guidelines, and registered in PROSPERO (CRD420251010774). Studies reporting AK treatment regiments and prognostic factors were included. After extracting the data from the included articles, the relevant aspects of the treatment and the prognostic factors were compared and summarized. **Results**: Sixty-one articles were included: nine were prospective, including 3 randomized controlled trials (RCTs), and fifty-two were retrospective. The findings suggest that therapeutic epithelial debridement (TED), followed by an association with biguanides, diamidines, and an antibacterial agent, is a strong initial treatment option. An adjunctive medical treatment with topical voriconazole 1% or oral miltefosine may also be considered. Surgical approaches were also assessed when the pharmaceutical therapy failed, with Deep Anterior Lamellar Keratoplasty (DALK) playing an important role in the cases without a deep stroma involvement. Early Therapeutic Penetrating Keratoplasty (TPK) should be used as a salvage therapy and Optical Penetrating Keratoplasty (OPK) should be used for rehabilitation purposes. Key prognostic factors include older age, delayed diagnosis, corticosteroid use before prompt diagnosis, poor initial best corrected visual acuity (BCVA), and AK stage at presentation. **Conclusions**: The initial treatment with TED, biguanides, and diamidines remains the foundation of treatment. Surgical options can be considered in advanced cases. An early diagnosis, age, and initial BCVA are prognosis factors that should be considered. Future research may focus on improvement of protocols and searching for novel agents.

## 1. Introduction

Acanthamoeba is an ubiquitous, free-living protozoan found in common environments such as water, soil, air, and dust [1]. These pathogens can cause a rare but severe sight-threatening corneal infection, which often leads to a poor prognosis [2,3,4,5]. Contact lens (CL) use has been reported as the most significant risk factor for AK, particularly in individuals with poor hygiene practices [6]. Additionally, environmental exposure, ocular trauma, pre-existent ocular surface diseases, and immunocompromised states are also important risk factors for AK [7,8]. The increasing prevalence of these risk factors, namely the rise in CL use, and the development of more accurate diagnostic tools for AK can potentially explain the increasing global frequency of AK [2,9,10]. The estimated global annual incidence of AK for 2023 is 23,561 cases, with a prevalence rate of about 2.9 cases per million people [3].

A key clinical characteristic of AK is severe ocular pain, which is disproportionate to the clinical signs in the ophthalmological examination. In the early stages of the disease, the epithelium and subepithelial layers are affected, often presenting with epithelial infiltrates, perineuritis, and pseudodendrites. In contrast, late-stage AK typically involves the stromal layer and it may also have extracorneal manifestations such as scleritis, iris atrophy, anterior synechiae, secondary glaucoma, and cataracts [11,12]. The classification for AK stage at presentation by Carnt et al. [13] has been increasingly used in AK studies, in which severity at presentation is divided into three categories: stage 1, corneal epitheliopathy only; stage 2, the presence of one or more of the following: corneal epithelial defects, perineural infiltrates or stromal infiltrates, in addition to stage 1 findings; and stage 3, the presence of a corneal stromal ring infiltrate in addition to one or more features of stage 2 disease. Nonetheless, the initial signs and symptoms are often non-specific, which can lead to a misdiagnosis, frequently of herpetic keratitis [12].

Diagnostic confirmation of AK can be achieved through a cultural analysis of corneal scrapings or through microscopic techniques [12]. However, these techniques have certain limitations that can prolong incubation time and delay diagnosis [14]. Therefore, polymerase chain reaction (PCR) is increasingly being utilized as it offers a faster response while ensuring good sensitivity [9]. In vivo confocal microscopy (IVCM) is also a powerful diagnostic tool, with a high sensitivity and specificity when performed by an experienced operator, without the need to wait for the culture and the microbiological analysis [12,15,16].

Establishing an effective treatment regimen remains challenging due to the small number of clinical trials available, the cysts resistance to the amebicides [10,11,17], and the variability in AK presentation [17]. Early intensive treatment is more effective since cysts have not yet fully matured. Currently, topical treatments with biguanides such as chlorhexidine (CHX) and polyhexamethylene biguanide (PHMB) are considered as first-line therapy for AK [18,19,20], with diamidines, such as propamidine isethionate and hexamidine, often used concurrently to enhance treatment efficacy [10,21]. Adjuvant therapies, such as neomycin, antifungals (voriconazole and itraconazole), and other amebicide agents (such as miltefosine), may play an essential role in improving the therapy response, particularly in advanced/refractory AK [11,12,22,23]. The use of corticosteroids (CCT) remains controversial. Some studies report an association between its use before the diagnosis and a poorer visual outcome [24,25], while others report decreased inflammation and pain when they are used after the start of an anti-amoebic therapy (AAT) [26]. Surgery may be required in cases of refractory/advanced AK. Therapeutic epithelial debridement (TED), therapeutic penetrating keratoplasty (TKP), and deep anterior lamellar keratoplasty (DALK) are some of the techniques used [12,17].

Prognostic clinical data for AK is still limited, but some studies describe the late diagnosis and low initial visual acuity as important factors to consider [27,28].

As AK becomes increasingly prevalent it is essential to improve our management of this ocular infection. Additionally, identifying the prognostic factors and addressing them could help mitigate complications and improve outcomes. Poor prognosis is still a significant reality in AK, given that early diagnosis and treatment are difficult to accomplish, and there are no definitive treatment guidelines for AK.

Therefore, this study aims to conduct a systematic review of the clinical data available, regarding the treatment protocols and prognosis factors for AK management. The purpose is to explore and help to develop an evidence-based algorithm, without disregarding prognosis factors that the physician should account for.

## 2. Materials and Methods

The primary aim of this literature review was to collect all currently available longitudinal data on AK therapeutic management and prognostic factors, in order to retrieve conclusions that can improve disease management. The Preferred Reporting Items for Systematic Reviews and Meta-Analyses (PRISMA) statement methodology was used in this review [29]. This review was registered in PROSPERO with the ID number CRD420251010774.

### 2.1. Search Strategy

A search strategy was performed on 22 July 2024 through three electronic databases, namely MEDLINE^®^ (via PubMed), SCOPUS^®^, and Web of Science^®^ using the following items based on three concepts:

1. Study type: “randomized controlled trial”, “RCT”, “cohort studies”, “follow-up studies”, “longitudinal studies”, “prospective studies” or “retrospective studies”.
2. Acanthamoeba: “Acanthamoeba”, “Ameba”, “Acanthamoeba Keratitis”, “Ameba Keratitis”, “Parasitic Keratitis”, “Acanthamoeba corneal infection”, “Ameba corneal infection”, “contact lens keratitis” or “contact lens associated keratitis”
3. AK-related parameters: “treatment”, “surgery”, “follow-up”, “complications”, “prognosis”, “management” or “therapy”.

The search query for each database can be found in Table S1 in the Supplementary Material.

### 2.2. Eligibility Criteria

Eligible studies fulfilled all the inclusion criteria listed below:

1. Studies with a sample size of at least 10 eyes with isolated Acanthamoeba corneal infection.
2. Outcome is one of the following AK-related parameters: type of treatment, need for surgery, type of surgery, follow-up, complications, prognosis or prognostic factors, and management strategies.

To ensure robust and significant results, we considered it necessary to include studies with a sample size of at least 10 eyes.

Exclusion criteria were as follows: case reports, reviews, studies performed in vitro/not clinical studies, meeting abstracts, animal studies, and studies not written in the English language.

### 2.3. Study Selection, Data Collection Process and Data Items

**Figure 1:**
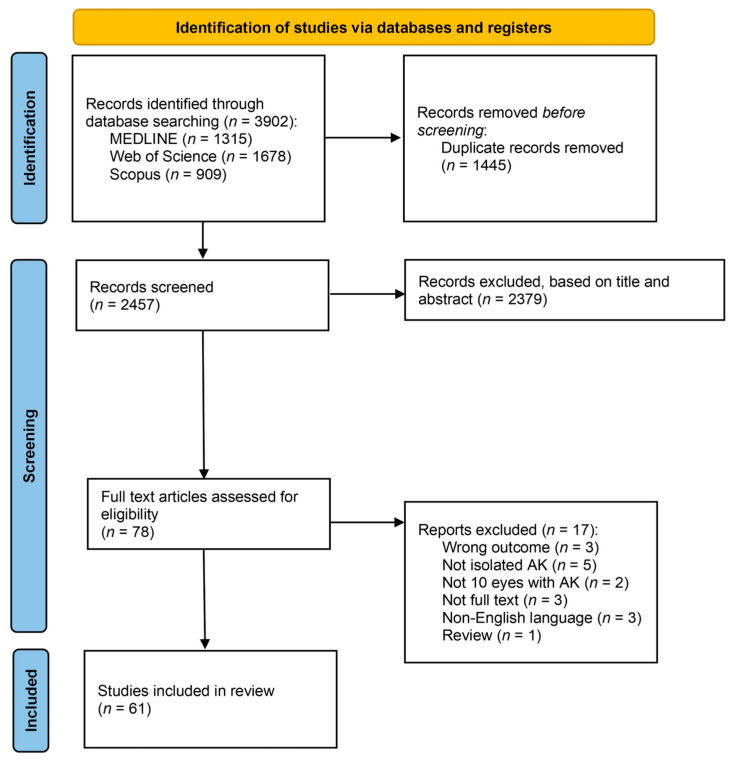
PRISMA 2020 flow diagram for new systematic reviews which included searches of databases and registers only [29].

Rayyan was used to initially remove duplicates, and Mendeley to manage the bibliography of the selected literature. After removing the duplicates from the identified articles in the three databases, study selection was carried out independently by two reviewers, initially through title/abstract screening (P.M.C. and M.M.) and subsequently through full-text reading (P.M.C. and M.M.). A third reviewer (R.V.M.) resolved disagreements at each phase (Figure 1).

Data extraction from the included studies was performed independently by two reviewers (P.M.C. and M.M.) using a purposely built internal online form. The following information was collected when available: authors, year of publication, country, study design, number of patients and eyes, follow-up time (months), medical therapy, surgical therapy, good and poor treatment outcomes, initial and final best corrected visual acuity (BCVA), improvement of BCVA, and good and poor prognosis factors (Table 1, Table 2, Table 3 and Table 4).

### 2.4. Study Risk of Bias Assessment

The quality of the articles was independently analyzed by two reviewers (M.M, P.C) using the National Institutes of Health (NIH) quality assessment tool for observational cohort and cross-sectional studies [30], the NIH quality assessment tool for case series studies [30], the NIH quality assessment tool for case–control studies [30], and the Cochrane risk of bias tool, version 6.5, 2024, for randomized control trials [31]. Traffic-light and summary plots were developed with robvis (visualization tool; Version: 0.3.0) [32].

## 3. Results

### 3.1. Study Selection

The search identified 3902 articles. After all duplicates were removed, 2457 articles remained, which were subject to title and abstract screening, considering the exclusion and inclusion criteria. This led to 78 studies. Afterward, each full text was read to ascertain eligibility. Excluded studies in this stage and the reasons for their exclusion are presented in Table S2. Three full-text articles were not found through online research, and the respective authors were contacted by email to request the full text, but no response was received. Ultimately, 61 articles were included and data extracted are presented in Figure 1.

### 3.2. Study Characteristics

A total of 61 articles were included. A summary of the included studies’ characteristics is presented in Table 1. Of the 61 studies, 9 were prospective [18,26,33,34,35,36,37,38] (including 3 RCTs [18,33,36]), and 52 studies were retrospective (including 2 case–control studies [24,39]). The articles were published between 1993 and 2024, and the studies were conducted in several countries.

Treatment strategies were assessed in several studies, including therapy with amebicides (biguanides and diamidines), antifungals (voriconazole), antiparasitic therapy (miltefosine), and the use of CCT before and after diagnosis of AK. Other medical treatments were considered as first-line options, such as the combination of propamidine isethionate 0.1% and neomycin-polymyxin B with gramicidin, as reported by Hargrave et al. (1999) [34]. Additionally, Caruso et al. (2020) studied a solution that contained CHX 0.02% and D-α-tocopheryl polyethylene glycol succinate (vitamin E TPGS or VE-TPGS) at 0.2% [35]. Surgical strategies discussed in studies include TED, TPK, OPK, and DALK. Ultimately, many articles addressed prognostic factors, especially the time until the start of treatment, stage of AK disease, use of CCT, age, and BCVA at presentation (Table 1, Table 2, Table 3 and Table 4).

**Table 1:** General characteristics of the included studies.

Article	Study Design	Population (Country; Age (Mean); Sex (M, F)(%))	No Patients/Eyes	Follow-Up (Months)
Pang et al. (2024) [40]	Retrospective cross-sectional	China, 41.5 y, M (55.3%), F (44.7%)	109/109, results and outcomes were not available for 1 patient	6.9
List et al. (2021) [41]	Retrospective cohort	Austria, 31 y, M (54.5%), F (45.5%)	42/44	N/A
Megha et al. (2020) [38]	Prospective case series	North India; 33 y; M (72.7%), F (27.3%)	11/11, 1 lost in follow-up	N/A
Jo et al. (2020) [42]	Retrospective cohort	Busan, South Korea; 21 y; M 12.5%, F 87.5%	16/19	5.365 (range of 1–20)
Musayeva et al. (2020) [22]	Retrospective case series	Germany; 41.7 y; M 42.9%, F 57.1%	28/28, 2 lost in follow-up	N/A
Papa et al. (2020) [43]	Retrospective cohort	UK (London); 13–33 y: 51.98% 34–76 y: 48.02%; M: 44.05%; F: 55.95%	227/227	At least 12 months after AAT
Lin et al. (2018) [44]	Retrospective interventional case series	Taiwan; 23.4 ± 11.1 (range 13–49) y; M (45.5%), F (54.5%)	22/24	12
McKelvie et al. (2018) [45]	Retrospective case series	New Zealand; 36 (15–66) y; M (44.2%); F (55.8%)	52/58	9
Lim et al. (2008) [18]	Prospective, double-masked, randomized comparative trial	London (Moorfields Eye Hospital); 31 y (IQR, 24 to 36 years); M (45.1%), F (52.94%)	55/56, only 51 eyes were analyzed in the study	PHMB group: 2.7 ± 2.0 CHX group: 3.0 ± 1.2
Wouters et al. (2022) [24]	Retrospective case–control	The Netherlands (Rotterdam) Control: 41 ± 17 y (15–70); M (58.5%); F (41.5%) Cases: 35 ± 17 y (11–74); M (46.43%), F (53.57%)	109/109	18 ± 25 (range 1–207)
Thulasi et al. (2021) [23]	Descriptive, retrospective multicenter case series	EUA 47.4 y (16 to 80) M (20%); F(80%)	15/15	7
Rahimi et al. (2015) [26]	Prospective interventional case series	Iran 21.5 ± 4.6 y M (12.9%), F (87.1%)	31/31	4
Robaei et al. (2014) [25]	Retrospective cohort	London, UK CCT before diagnosis: 40.3 y; M (48.28%), F (51.72%) Not-CCT before diagnosis: 33.6 y; M (44.83%), F (55.17%)	174/174	CCT before diagnosis: 18.5 (6.5–44) Not-CCT before: 7.5 (4.5–24)
Bagga et al. (2021) [33]	Prospective, randomized, double-masked pilot clinical trial	India; 39.8 ± 14.3 y; M (61.1%), F (38.9%)	23/23, only 18 patients completed the study	2.8 ± 2.4
Azuara-Blanco et al. (1997) [46]	Retrospective case series	UK 30.0 ± 7.4 y (19–40) M (60%), F (40%)	10/10	7.3 (range 4–15)
Duguid et al. (1997) [21]	Retrospective case series	London, UK 32 y (16–64) M (57.1%), F (42.9%)	105/111	N/A
Hargrave et al. (1999) [34]	Prospective, multicenter noncomparative case series	EUA 33 y (18–72) N/A	83/87, only 60 eyes had their data analyzed for treatment efficacy and safety	15
Carnt et al. (2016) [47]	Retrospective cohort.	London, UK 33 y (15–76) M (47.3%), F (54.0%)	196/196, only 129 included in the multivariable analysis; eyes with scleritis or hypopyon at the start of AAT were excluded (as these conditions were strongly associated with previous corticosteroid use)	N/A
Höllhumer et al. (2020) [48]	Retrospective case review	Sydney, Australia 39 y (14–89) M (40%), F (60%)	52/52	33
Caruso et al. (2020) [35]	Prospective interventional case series study.	Naples, Italy 27.03 ± 10.61 y M (31.04%), F (68.96%)	29/29	At least 3 months after completion of treatment
Dart et al. (2024) [36]	Prospective, randomized, double-masked, active-controlled, multicenter phase 3 clinical trial	UK, Italy and Poland 36.7 ± 13.8 y (15–73) M (41.7%), F (58.3%)	135/135, 127 full-analysis subset; 134 in the safety analysis subset	12
Blaser et al. (2024) [49]	Retrospective, single-center chart review	Switzerland 33 y (13–90) M (40.4%), F (59.6%)	52/59, 46 were treated	N/A
Tew et al. (2020) [50]	Retrospective case series	Taiwan, 12–56 (28 y) M (50%), F (50%)	107/107, 10 eyes with AK	Postoperative follow-up period was at least 1 month in all patients
Chen et al. (2004) [51]	Retrospective interventional case series	Taiwan 57.6 y (17–84) N/A	108/108, 15/15 with AK	12
Laurik et al. (2019) [52]	Retrospective observational cohort	Germany 39.6 ± 13.3 y N/A	23/23	27 ± 19.4
Liu et al. (2020) [53]	Retrospective case series	Taiwan 27.1 ± 1.5 y (12–65) M (45.2%), F (54.8%)	62/62	N/A
Kitzmann et al. (2009) [54]	Retrospective, nonrandomized, comparative, interventional case series	USA TPK: 40 y; OPK: 30 y (13–72) M (40%), F (60%)	30/31	N/A
Qi et al. (2024) [55]	Retrospective cohort	China BB-DALK Group: 44.56 ± 15.5 y; M: 63.0% (17/27), F: 37.0% (10/27) PKP Group: 48.79 ± 15.7 y M: 45.8% (11/24), F: 54.2% (13/24)	51/51	BB-DALK 26.78 ± 21.11 (range, 0–78) PKP 27.29 ± 21.23 (range, 0–79)
Wang et al. (2023) [56]	Retrospective case series	China 45.54 ± 11.78 y (24–66) M (61.5%); F (38.5%)	13/13	21.31 ± 19.59 (range, 12–82)
Zhang et al. (2023) [57]	Retrospective cohort	China 44.6 ± 12.4 y M (56.9%), F (43.1%)	58/59 with stage 3 AK requiring keratoplasty	PK: 6.2 ± 3.7 LK: 6.2 ± 7.5
Roozbahani et al. (2019) [58]	Retrospective case series	USA 49 ± 18 y (16–73 y) M (33%), F (66%)	63/63	3 months after ending therapy
Sabatino et al. (2016) [59]	Retrospective, noncomparative case series	Italy 24.5 ± 7 y (15–37) M (45%), F (55%)	11/11	25 ± 10 (range, 12–39)
Robaei et al. (2015) [60]	Retrospective case series	UK OPK: 44.3 ± 16.6 y; M (50%), F (50%) TPK: 43.0 ± 12.1 y; M (61.5%), F (38.5%)	196/50	62 ± 3
Bagga et al. (2020) [61]	Retrospective cohort	India 38.7 ± 8.6 y (25–56) M (50%), F (50%)	23/23, 10 with advanced AK (infiltrate ≥ 8 mm)	5 (IQR, 1.4–11.4)
Thebpatiphat et al. (2007) [62]	Retrospective case series	USA 38 y (13–75) M (55%), F (45%)	20/20	N/A
Butler et al. (2005) [63]	Retrospective case series	Australia 38.9 ± 14.0 y M (45%), F (55%)	20/20	24.8 ± 21.5 (1–72)
Chin et al. (2015) [64]	Retrospective case series	Hong Kong 24.1 ± 8.01 y (13 to 38 y) M (30.8%), F (69.2%)	13/15	N/A
Alfonso-Muñoz et al. (2018) [65]	Retrospective case series	Spain 28.4 y M (60%), F (40%)	10/10 Superficial stroma: 3/10 (30%) Deep stroma (ring infiltrates or corneal abscesses): 7/10 (70%)	12
Kaiserman et al. (2012) [66]	Retrospective cohort	Canada 34.2 ± 13.2 y M (51.2%), F (48.8%)	41/42	19.7 ± 21.0 (median: 13)
Ikeda et al. (2012) [67]	Retrospective cross-sectional	Japan 26.4 ± 1.7 y M (39.3%), F (60.7%)	103/104, 28/29 with AK	N/A
Patel et al. (2010) [68]	Retrospective case series	New Zealand 40 ± 13 y M (40%), F (60%)	25/25, 1 lost in follow up	N/A
Shimmura-Tomita et al. (2018) [69]	Retrospective comparative case series	Japan Severe group: 37 y Mild group: 24 y M (50%), F (50%)	10/10	N/A
Zhang et al. (2014) [70]	Retrospective cohort	China 44 ± 12 y M (62%), F (38%)	29/29	N/A
Elmer et al. (2008) [39]	Retrospective case–control	USA 28.7 ± 14.9 y M (52.8%), F (47.2%)	65/72 Cases: ≥0.1 logMAR or PKP Control: ≤0.1 logMAR	At least 3 months post-treatment completion
Radford et al. (1998) [71]	Multicenter retrospective cross-sectional	England 31.5 y (4 to 64) M (56%), F (44%)	243/259	6
Vilares-Morgado et al. (2024) [28]	Retrospective, single-center, longitudinal	Portugal 35.7 ± 13.8 y (14 to 66) M (28.7%), F (71.3%)	46/51 BCVA final < 1 logMAR group: 31 (60.8%) BCVA final ≥ 1 logMAR group: 20 (39.2%)	39 ± 30.2 (14–120)
Randag et al. (2019) [72]	Retrospective case series, multicenter nationwide survey	Netherlands 34 y (11 to 75) M (37.5%), F (62.5%)	224/224	11.6 ± 27.4
Bacon et al. (1993) [73]	Retrospective case series	UK; N/A; N/A	72/77, 4 lost to follow up	3
G D’Aversa et al. (1995) [74]	Retrospective case series	USA; N/A; N/A	12/14	N/A
Claerhout et al. (2004) [75]	Retrospective case series	Belgium 32 y (19 to 64) M (71%), F (29%)	14/14 Early treatment ≤18 days of symptom onset: 6/14 Late treatment >18 days: 8/14	34 (range 1–63)
Chew et al. (2011) [76]	Retrospective case series	USA 34 y (12 to 75) M (47%), F (53%)	59/59	11.2 (range: 0.4–35.1)
Bouheraoua et al. (2013) [27]	Retrospective noncomparative interventional case series	France 43 y (14 to 90) M (28%), F (72%)	42/44	10 (1–49)
Carnt et al. (2018) [13]	Retrospective cohort	UK; N/A; N/A	194/194	Bad Outcomes group: 32 (IQR: 15–58) Good Outcomes group: 7 (IQR: 4–1)
Chopra et al. (2020) [77]	Retrospective cohort	UK 41.5 ± 15.7 y (20 to 81) M (44.6%), F (55.4%)	157/157	At least 3 months after stopping treatment
Bonini et al. (2021) [78]	Retrospective case series	Italy 30 ± 13 y N/A	35/40	146.4 ± 66
Rahimi et al. (2014) [37]	Prospective intervencional case series	Iran 23 ± 6 y (14–36) M (16%), F (84%)	25/27	At least 6 months after ending treatment
Yamazoe et al. (2012) [79]	Retrospective case series	Japan 29.4 ± 8.9 y (16–49) M (71.4%), F (28.6%)	34/35 Group 1: final VA of ≤ 0.10 logMar (22 eyes) Group 2: >0.10 logMar (13 eyes)	Group 1: Median 6.7 (IQR 5.2–11.8) Group 2:Median 9.5 (IQR 4.7–42.5)
Arnalich-Montiel et al. (2014) [80]	Prospective cohort	Spain; N/A; N/A	17/17	Median: 6 months
Park et al. (1997) [81]	Retrospective case series	USA; Steroid-treated: 36.2 14.0; M (50%), F (50%). Non-steroid treated: 27.8 9.0; M (33.3%), F (67.6%)	38/38	N/A
Landeo et al. (2015) [82]	Retrospective case series	Peru; 24.9 y; M (50%), F (50%)	14/14 early treatment (<30 days): 5/14 late treatment (≥30 days): 9/14	N/A
Sun et al. (2006) [83]	Retrospective, noncomparative, interventional case series	China; 26 y (ranged from 12 to 50); M (60%), F (40%)	20/20	8.8 (range, 2.5–25.4)

Abreviations: y (year); M (Male); F (Female); PHMB (polyhexamethylene biguanide); CHX (Chlorexidine); CCT (Corticosteroid); N/A (Not Applicable); AAT (Anti-Amebic Therapy); AK (Acathamoeba Keratitis); BB-DALK (Big-Bubble Deep Anterior Lamelar Keratoplasty); PKP (Penetrating Keratoplasty); OPK (Optical Keratoplasty); TPK (Therapeutic Keratoplasty).

**Table 2:** Medical and Surgical Therapies Reported by Included Studies.

Article	No Patients/Eyes	Medical Therapy, *n* (%)	Surgical Therapy, *n* (%)
Pang et al. (2024) [40]	109/109, Results and outcomes were not available for 1 patient	CHX 0.02% + PHMB 0.02%, 109 (100%) (initially used hourly for the first week and tapered slowly over the following four weeks to dosing four times a day). Maintenance therapy: 0.02% CHX four times a day for three to six months.	N/A
List et al. (2021) [41]	42/44	Biguanide (0.02%), 42 (95.5%); Diamidine (0.1%), 40 (90.9%)	N/A
Megha et al. (2020) [38]	11/11, 1 lost in follow-up	PHMB (0.02%), 10 (100%) (half hourly for 1 week, then hourly for 1 week and then gradually tapered according to the response)	N/A
Jo et al. (2020) [42]	16/19	PHMB 0.02%, 9 (47.4%) CHX 0.02%, 5 (26.3%) PHMB + CHX, 5 (26.3%)	N/A
Musayeva et al. (2020) [22]	28/28, 2 lost in follow-up	PHMB 0.02% + propamidine 0.1% + voriconazole 0.1%: 26 (100%); 24 h/36 h: every 15 to 30 min in an alternating fashion during day and night. Application was then reduced to 4 times daily each in an alternating fashion during the day. Dexamethasone 0.13% after 24/36 h to reduce inflammation and corneal scarring	N/A
Papa et al. (2020) [43]	227/227	CHX 0.02% or PHMB 0.02% Propamidine 0.1% or hexamidine 0.1% (Monotherapy or association between drugs)	N/A
Lin et al. (2018) [44]	22/24	Ethanol pretreatment (30 s before Epithelial debridment) + PHMB (0.02%) + propamidine (0.1%), 24 (100%)	TED, 24 (100%)
McKelvie et al. (2018) [45]	52/58	Biguanide (PHMB 0.02% or CHX 0.02%) + Propamidine 0.1%, 58 (100%) Empiric topical CCT, 6 (10.3%)	N/A
Lim et al. (2008) [18]	55/56, only 51 eyes were analyzed in the study	PHMB 0.02%, 23 (45%) CHX 0.02%, 28 (55%)	N/A
Wouters et al. (2022) [24]	109/109	Cases: CCT before AAT, 56 (51%) Controls: 53 (49%)	N/A
Thulasi et al. (2021) [23]	15/15	Oral Miltefosine (50 mg once or twice a day) as adjunctive therapy, 15 (100%)	N/A
Rahimi et al. (2015) [26]	31/31	Monotherapy with CHX 0.02%, 31 (100%) Topical CCT(at least 2 weeks after treatment with chlorhexidine), 26 (83.9%) CCT before diagnosis: 12 (38%)	N/A
Robaei et al. (2014) [25]	174/174	CTT before diagnosis: 87 (50%) (63 (73.3%) misdiagnosed as herpetic keratitis) (*p* < 0.001) No CCT before diagnosis: 87 (50%) (28 (32.2%) misdiagnosed as herpetic keratitis)(*p* < 0.001)	N/A
Bagga et al. (2021) [33]	23/23, only 18 patients completed the study	VZ group: Topical 1% voriconazole monotherapy: 8 (44.4%) BG group: PHMB 0.02% + CHX 0.02%: 10 (55.5%) Both hourly during the day and every 2 h at night for 1 week, followed by gradual tapering.	N/A
Azuara-Blanco et al. (1997) [46]	10/10	PHMB 0.02% + propamidine 0.1%: 9 (90%) CHX 0.02% + propamidine: 1 (10%) (later switched to PHMB); Polymixin B sulfate and bacitracin zinc, 10 (100%) Prednisolone acetate 0.5%, 2–4 times daily, 10 (100%)	2 OPK for visual rehabilitation due to scarring, 2 (20%)
Duguid et al. (1997) [21]	105/111	PHMB 0.02% + propamidine 0.1%, 111 (100%) Applied hourly, day and night for 2–3 days initially, then reduced based on response. Therapy continued for 6–8 weeks after resolution of inflammation.	N/A
Hargrave et al. (1999) [34]	83/87, only 60 eyes had their data analyzed for treatment efficacy and safety	Propamidine isethionate 0.1% + neomycin-polymyxin B-gramicidin, 87 (100%)	N/A
Carnt et al. (2016) [47]	196/196, only 129 were included in the multivariable analysis	CCT after start of AAT: 73 (56.6%) No CCT: 56 (43.4%)	N/A
Höllhumer et al. (2020) [48]	52/52	PHMB, CHX, Propamidine (28 (54%) used a triple combination; 23 (44%) used dual therapy; 1 (2%) monotherapy) Adjunctive therapy: Oral voriconazole: 9 (17%)	N/A
Caruso et al. (2020) [35]	29/29	CHX 0.02% and VE-TPGS 0.2%: 29 (100%)	N/A
Dart et al. (2024) [36]	135/135, 127 in the full-analysis subset; 134 in the safety analysis subset	PHMB 0.08%: 66 (51.96%) PHMB 0.02% + propamidine: 61 (48.03%) Every hour for the first 19 days. Tapered to four times daily over the next months.	N/A
Blaser et al. (2024) [49]	52/59, 46 were treated	Propamidine 0.1% + PHMB 0.02% hourly (48 h day/night) → hourly (72 h daytime) → tapered (~3×/day) for 12 months Dexamethasone 0.1% after 4 weeks	Initial TED, 46 (100%)
Tew et al. (2020) [50]	107/107, 10 eyes with AK	N/A	TPK: 10 (100%)
Chen et al. (2004) [51]	108/108, 15/15 with AK	N/A	PKP after medical treatment failure: 15 (100%)
Laurik et al. (2019) [52]	23/23	PHMB (0.02%) + propamidine (0.1%) + Neomicine (0.02%) (100%)	KP after intensive topical triple therapy failure (100%) Early PK (<5.3 months of symptom onset): 14/23 (61%) Delayed PK (after 5.3 months): 9/23 (39%)
Liu et al. (2020) [53]	62/62	Medically treated 40 (64.5%)	OPK: 10 (25%) TPK: 17 (77.3%)
Kitzmann et al. (2009) [54]	30/31	N/A	TKP: 22 (71%); OPK: 9 (29%) Ring infiltrate (TPK 82% vs. OPK 0%, *p* = 0.001) or epithelial defect (TPK 64% vs. OPK 22%, *p* = 0.05)
Qi et al. (2024) [55]	51/51	N/A	PKP: 24 (47%) (Cases with higher incidence of ring infiltrates, endothelial plaques, hypopyon, uveitis, and glaucoma (*p* = 0.007)) BB-DALK: 27 (53%) (Lower percentage of stage 3 disease (*p* = 0.003))
Wang et al. (2023) [56]	13/13	N/A	Elliptical DALK: 13 (100%)
Zhang et al. (2023) [57]	58/59 with stage 3 AK requiring keratoplasty	N/A	PKP: 36 (61%) LKP: 23 (39%)
Roozbahani et al. (2019) [58]	63/63	PHMB 0.02% + CHX 0.02% (or combined with propamidine) 51 (81%); neoadjuvant therapy: voriconazole, itraconazole or neomycin	TPK, 12 (19%)
Sabatino et al. (2016) [59]	11/11	N/A	Early therapeutic DALK (manual dissection or BB) (within 30 to 60 days from the onset of symptoms), 11(100%)
Robaei et al. (2015) [60]	196/50	N/A	TKP: 26 (52%); OKP: 24 (48%)
Bagga et al. (2020) [61]	23/23, 10 with advanced AK (infiltrate ≥8 mm)	PHMB 0.02% + CHX 0.02%; prednisolone acetate 1% started after infection control	DALK: 23 (100%) (10 (43%) advanced AK/ 13 (57%) less severe AK)
Thebpatiphat et al. (2007) [62]	20/20	N/A	N/A
Butler et al. (2005) [63]	20/20	PHMB 0.02% + propamidine 0.1%, 20 (100%) CHX 0.02%: 9 (45%) Neosporin^®^: 8 (40%)	N/A
Chin et al. (2015) [64]	13/15	AAT:15 (100%) CCT: 9 (60%)	N/A
Alfonso-Muñoz et al. (2018) [65]	10/10, superficial stroma: 3/10 (30%) deep stroma (ring infiltrates or corneal abscesses): 7/10 (70%)	CHX 0.02% + Propamidine 0.1%: 10 (100%) Adjunctive treatment: polymyxin B sulfate, neomycin sulfate and gramicidin: 3 (30%) oral antifungal treatment (itraconazole or fluconazole): 4 (40%) CCT: 6 (60%) due to pain and inflammation (average of 53 days after beginning the AAT)	N/A
Kaiserman et al. (2012) [66]	41/42	PHMB 0.02%: 37 (88.1%) Propamidine 0.1%: 20 (47.6%) CHX 0.02%: 13 (31%) Neomycin, Polymyxin B, Gramicidin (Neosporin^®^): 32 (76.2%) Combination Therapy: 85.7% of patients received combination therapy, mostly with three agents (57.1%) Topical CCT: 26 (61.9%)	KP: 7 (22.6%)
Ikeda et al. (2012) [67]	103/104, 28/29 with AK	N/A	N/A
Patel et al. (2010) [68]	25/25, 1 lost in follow up	PHMB 0.02% or CHX 0.02% + Propamidine CCT before diagnosis: 14 (56%)	N/A
Shimmura-Tomita et al. (2018) [69]	10/10	N/A	N/A
Zhang et al. (2014) [70]	29/29	N/A	N/A
Elmer et al. (2008) [39]	65/72	N/A	N/A
Radford et al. (1998) [71]	243/259	Propamidina + PHMB: 108 (50%). Propamidina + Neomicina + PHMB: 31 (14%). Propamidina + Clorexidina: 15 (7%). (197 (90%) were treated with PHMB and/or CHX)	N/A
Vilares-Morgado et al. (2024) [28]	46/51 BCVA final < 1 logMAR group: 31 (60.8%) BCVA final ≥ 1 logMAR group: 20 (39.2%)	CHX (0.02%): 1 (2%) Propamidine (0.1%): 6 (12%) CHX + Propamidine: 41 (79%) Additional therapy: Oral Voriconazole: 1 (2%) Oral Miltefosine: 2 (4%)	TED: 22 (43%) KP: 18 (35%) for corneal perforation or treatment failure Evisceration/Enucleation: 12 (23.5%)
Randag et al. (2019) [72]	224/224	CHX monotherapy:220 (98%) CHX + PHMB/Propamidine:184 (82%) CCT after diagnosis: 92 (41%)	N/A
Bacon et al. (1993) [73]	72/77, 4 lost to follow up	Propamidine + neomycin, 29 (39.7%); Propamidine + PHMB, 13 (17.8%); Propamidine + neomycin + PHMB, 12(16.4%); broader combinations in 22 eyes (30.1%) included paromomycin, itraconazole, and others.	34 KP in 23 eyes: TPK: 21 in inflamed eyes OPK: 13 in uninflamed eyes
G D’Aversa et al. (1995) [74]	12/14	Propamidine isethionate + neomycin sulfate + clotrimazole, 11 (79%); neomycin-gramicidin-polymyxin, 2 (14%); broad-spectrum agents, 1 (7%)	TKP: 3 (21%) (1 for bacterial superinfection, 1 for large descemetocele, 1 after failed medical treatment)
Claerhout et al. (2004) [75]	14/14, early treatment ≤18 days of symptom onset: 6/14 late treatment >18 days: 8/14	Propamidine + chlorhexidine or PHMB 14 (100%)	N/A
Chew et al. (2011) [76]	59/59	PHMB 0.02%, Propamidine, and antibiotics (100%); oral antifungals (itraconazole/voriconazole) in severe cases; 61% treated with corticosteroids after AK diagnosis;	N/A
Bouheraoua et al. (2013) [27]	42/44	Hexamidine 0.1% + PHMB 0.02%: 44 (100%); Oral itraconazole in severe cases: 6/44 (14%); CCT after 2 weeks starting AAT: (66%).	AMT: 8 (18%) PKP: 4 (9%) Evisceration: 2 (4%)
Carnt et al. (2018) [13]	194/194	PHMB 0.02%, 184 (95%); Adjunctive agents including CHX and propamidine. Corticosteroids pre-AAT: 56 (29%)	N/A
Chopra et al. (2020) [77]	157/157	N/A	N/A
Bonini et al. (2021) [78]	35/40	propamidine isethionate 0.1% and 0.02% PHMB: (100%)	N/A
Rahimi et al. (2014) [37]	25/27	PHMB 0.02%: 27 (100%)	N/A
Yamazoe et al. (2012) [79]	34/35 Group 1: final VA of ≤0.10 logMar (22 eyes) Group 2: >0.10 logMar (13 eyes)	CHX 0.02% hourly, gradually tapered	Group 2: KP: 3 (23%) (2 PKP, 1 DALK)
Arnalich-Montiel et al. (2014) [80]	17/17	N/A	N/A
Park et al. (1997) [81]	38/38	CCT, 19 Non-CCT, 17 CCT before diagnosis, 9	N/A
Landeo et al. (2015) [82]	14/14	PHMB 0.02% and Propamidine 0.1%	N/A
Sun et al. (2006) [83]	20/20	CHX 0.02% + Neomycin (0.5%) Itraconazole in severe cases, 15 (75%)	N/A

Abreviations: N/A, Not Applicable; CHX, Chlorexidine; PHMB, polyhexamethylene biguanide; CCT, Corticosteroid; OPK, Optical Keratoplasty; PKP, Penetrating Keratoplasty; KP, Keratoplasty; TED, Therapeutic Epithelial Debridement; AK, Acanthamoeba Keratitis; AAT, Anti-Amebic Therapy; BB-DALK, Big-Bubble Deep Anterior Lamelar Keratoplasty, VE-TPGS, D-α-tocopheryl polyethylene glycol succinate (vitamin E TPGS); BCVA, Best Corrected Visual Acuity.

**Table 3:** Prognostic factors reported by included studies.

Article	No Patients/Eyes	Good Prognosis	Poor Prognosis
Pang et al. (2024) [40]	109/109, results and outcomes were not available for 1 patient	T4C genotype (*p* = 0.040)	T4D genotype (*p* = 0.013)
List et al. (2021) [41]	42/44	N/A	Greater time until diagnosis (*p* = 0.004); CCT use (OR = 7.33, 0.22–4.15); Older age (OR = 1.06); Poor BCVA at initial presentation (OR = 9.59); Increased follow-up time (OR = 1.02); Duration of symptoms (OR = 1.50).
Megha et al. (2020) [38]	11/11, 1 lost in follow-up	N/A	N/A
Jo et al. (2020) [42]	16/19	Early diagnosis of AK (*p* = 0.638)	N/A
Musayeva et al. (2020) [22]	28/28, 2 lost in follow-up	N/A	N/A
Papa et al. (2020) [43]	227/227	N/A	N/A
Lin et al. (2018) [44]	22/24	N/A	Symptom onset to treatment (37.3 days, mean,) (*p* = 0.020); RGP lenses: worse initial BCVA (*p* = 0.02) but more improvement in vision (*p* = 0.01).
McKelvie et al. (2018) [45]	52/58	N/A	Empirical CCT: associated with diagnostic delay 47 days (range 15–154 days): worse final BCVA ranging from 0.60 to PL
Lim et al. (2008) [18]	55/56, only 51 eyes were analyzed in the study	N/A	Delay in treatment (*p* = 0.07) Median delay (21 days): all eyes successfully treated Median delay (56 days): all eyes unsuccessfully treated
Wouters et al. (2022) [24]	109/109	N/A	CCT use prior to diagnosis (OR: 4.3 (95%CI (1.7–11.0)) (*p* = 0.002)
Thulasi et al. (2021) [23]	15/15	N/A	N/A
Rahimi et al. (2015) [26]	31/31	N/A	VA > 0.1: Symptoms duration ≥21 days before diagnosis (*p* = 0.165) CCT use before diagnosis (*p* = 0.29) First BCVA ≤0.3 logMar (*p* = 0.29) Corneal stromal involvement at diagnosis (*p* = 0.05)
Robaei et al. (2014) [25]	174/174	N/A	Older patients (≥60 years) (OR 8.97, 95% CI: 2.13–37.79)
Bagga et al. (2021) [33]	23/23, only 18 patients completed the study	N/A	N/A
Azuara-Blanco et al. (1997) [46]	10/10	Early diagnosis (<4 weeks of symptoms); Adjunctive CCT with anti-amoebic coverage.	N/A
Duguid et al. (1997) [21]	105/111	Early diagnosis (<28 days, *p* = 0.005); effective combined therapy with PHMB and propamidine	Delayed diagnosis (>2 months, *p* < 0.05) Secondary glaucoma Bacterial co-infections
Hargrave et al. (1999) [34]	83/87, only 60 eyes had their data analyzed for treatment efficacy and safety	Strict adherence to protocol; avoidance of premature surgical interventions.	N/A
Carnt et al. (2016) [47]	196/196, only 129 included in the multivariable analysis	N/A	CCT use before AAT (OR = 3.85, *p* = 0.012); Stage 3 AK; (OR = 5.89; *p* = 0.032). Older age > 33 years (OR = 4.02, *p* = 0.007).
Höllhumer et al. (2020) [48]	52/52	N/A	Treatment >21 days: mean VA of 0.86 ± 0.98 (*p* = 0.2) Stage 3: *p* = 0.04
Caruso et al. (2020) [35]	29/29	N/A	N/A
Dart et al. (2024) [36]	135/135, 127 in the full-analysis subset; 134 in the safety analysis subset	N/A	N/A
Blaser et al. (2024) [49]	52/59, 46 were treated	N/A	N/A
Tew et al. (2020) [50]	107/107, 10 eyes with AK	N/A	Delay in diagnosis: eight out of 10 (80%) of whom six had previously received CCT (1 enucleation)
Chen et al. (2004) [51]	108/108, 15/15 with AK	Early intervention with PKP when medical treatment fails; Smaller graft sizes had better outcomes (<8.5 mm)	Delayed diagnosis (>2 months); Poor contact lens hygiene; Scleral extension of infection.
Laurik et al. (2019) [52]	23/23	N/A	N/A
Liu et al. (2020) [53]	62/62	N/A	Presence of ring infiltrate (with versus without): Initial VA: 1.51 ± 0.17 versus 1.71 ± 0.1, *p* = 0.2; Final VA: 0.41 ± 0.1 versus 1.17 ± 0.2, *p* = 0.002; Presence of complications (glaucoma, recurrence, dilated pupil/iris atrophy, graft rejection and graft failure) (*p =* 0.012)
Kitzmann et al. (2009) [54]	30/31	N/A	N/A
Qi et al. (2024) [55]	51/51	N/A	N/A
Wang et al. (2023) [56]	13/13	N/A	N/A
Zhang et al. (2023) [57]	58/59 with stage 3 AK requiring keratoplasty	N/A	CCT use before diagnosis (*p* = 0.040) and hypopyon (*p* = 0.009) were risk factors for recurrence after LK
Roozbahani et al. (2019) [58]	63/63	N/A	Therapy 25 days after symptoms (*p* = 0.041); Poorer presenting vision BCVA ≥ 1 logMAR unit (*p* = 0.002)
Sabatino et al. (2016) [59]	11/11	N/A	N/A
Robaei et al. (2015) [60]	196/50	N/A	Age > 70 (*p* = 0.070)
Bagga et al. (2020) [61]	23/23, 10: Advanced AK (infiltrate ≥8 mm)	N/A	Advanced keratitis with infiltrate ≥8 mm, posterior stromal involvement
Thebpatiphat et al. (2007) [62]	20/20	Superficial Cases: dendritiform keratitis or radial keratoneuritis (BCVA better than 0.18 logMAR units at 3 months) *p* = 0.00008 Early diagnosis statistically significantly shorter in the group with superficial pathology (*p* = 0.03)	Stage 3 AK and BCVA less than finger counting; *p* = 0.00008
Butler et al. (2005) [63]	20/20	N/A	Delayed diagnosis correlated to a higher recurrence (>1 month, *p* < 0.05); Poor contact lens hygiene
Chin et al. (2015) [64]	13/15	N/A	Delayed treatment (>30 days) (early (0.4 ± 0.9 logMAR units) vs. Late (1 ± 1.35 logMAR units); *p* = 0.125 CCT use before diagnosis (*p* = 0.367);
Alfonso-Muñoz et al. (2018) [65]	10/10 Superficial stroma: 3/10 (30%) Deep stroma (ring infiltrates or corneal abscesses): 7/10 (70%)	N/A	Deep stromal disease: OR: 10.27 (IC 95%): 2.91–36.17 Time until diagnosis: Superficial group (19 days); Deep group (56 days); *p* < 0.05
Kaiserman et al. (2012) [66]	41/42	Neuritis (*p* = 0.04) and pseudodendrites (*p =* 0.05) Good initial visual acuity (*p* = 0.002) Infections related to swimming (*p* = 0.01) Absence of an epithelial defect (*p* = 0.03) Having been treated with chlorhexidine (*p* = 0.05) Not having received CCT (*p* = 0.003)	Treated with topical CCT (*p* = 0.04) Epithelial defect on presentation (*p* = 0.0006) Longer-length of treatment (9.4 ± 3.7 months vs. 7.1± 2.9 months, *p* = 0.03) Neosporin^®^ prolongs the time of treatment (*p* = 0.03)
Ikeda et al. (2012) [67]	103/104, 28/29 with AK	N/A	Higher Acanthamoeba DNA copy numbers (OR per category, 3.48; 95% CI, 1.04 –111.63, *p* = 0.05) Advanced AK stage (OR:2.8 per stage increase (95% CI, 1.07–7.30, P0.05))
Patel et al. (2010) [68]	25/25, 1 lost in follow up	Early diagnosis of AK (<21 days)	Late diagnostic (>21 days): All surgical interventions occurred in this group (n = 6) Use CCT before diagnosis: 64% (9/14) were in the late diagnosis group
Shimmura-Tomita et al. (2018) [69]	10/10	N/A	Older age (*p* = 0.04) CCT use before diagnosis (100% (severe stage) vs. 67% (mild stage)) Keratoprecipitates (*p* = 0.01)
Zhang et al. (2014) [70]	29/29	N/A	Late-disease stage on presentation (deep stromal keratitis, ring infiltrate or extracorneal complications): OR 10.50 (95% CI (1.07–103.51) (*p* = 0.044) (*p* = 0.154) Deep location of cysts ≥ 250 µm: OR: 11.38 (95% CI (1.17–110.42) *p* = 0.036; (*p* = 0.215) Clusters or chains of cysts observed with IVCM: OR:14.86 (95%CI (1.53–144.2) *p* = 0.020
Elmer et al. (2008) [39]	65/72	N/A	Deep stromal disease or ring infiltrate: OR: 10.27 (2.91–36.17); *p* < 0.001 Duration between symptom onset and UIC presentation (>3 weeks): OR:2.55 (0.83–7.88); *p =* 0.10 Continued post-diagnosis steroid use: OR: 17.00 (95% CI, 3.19–90.66) (*p* = 0.02)
Radford et al. (1998) [71]	243/259	Early diagnosis of AK (<30 days) (170 eyes) (*p* < 0.01)	Late diagnosis of AK (>30 days) (67 eyes) (*p* < 0.01) Non-CL wearers (18 patients): delayed diagnosis and poorer visual outcomes compared to CL wearers, with only 10/18 (56%) achieving ≤ 0.30 LogMAR units
Vilares-Morgado et al. (2024) [28]	46/51 BCVA final < 1 logMAR group: 31 (60.8%) BCVA final ≥ 1 logMAR group: 20 (39.2%)	Early diagnosis (≤14 days after symptom onset): *p* = 0.004 (OR 19.78; 95% CI 2.07–189.11; *p* = 0.010) Epithelial debridment: (OR 19.02; 95% CI 3.27–110.57; *p* = 0.001) Better initial BCVA (0.8 ± 0.7 logMAR units vs. 1.3 ± 0.9 logMAR units; *p* = 0.047)	Late diagnosis (>14 days from symptom onset): BCVA > 1: 31 (54.8%); BCVA ≥ 1: 17 (94.4%)
Randag et al. (2019) [72]	224/224	N/A	CCT use before diagnosis increased failure: OR: 3.308 95%(1.375–7.963); Advanced disease stage (Stage 3): OR: 3.847 95%(1.544–9.584) Advanced age: OR 1.052 95% (1.029–1.075)
Bacon et al. (1993) [73]	72/77, 4 lost to follow up	Early diagnosis (<1 month) (*p* < 0.001), smaller ulcer size at presentation, and absence of microbial co-infection	Late diagnosis (>2 months), glaucoma, microbial co-infections, resistant strains, prolonged steroid use prior to correct diagnosis. TPK in inflamed eyes: Graft survival probability at 61 months: inflamed = 35%, uninflamed = 69%; OR = 7.34, *p* < 0.001;
G D’Aversa et al. (1995) [74]	12/14	Early diagnosis (<1 month of symptoms, *p* < 0.05); Absence of corticosteroid use	Late diagnosis (>5 months, *p* < 0.01); Bacterial superinfections contributed to treatment failures.
Claerhout et al. (2004) [75]	14/14 Early Treatment ≤18 days of symptom onset: 6/14 Late Treatment >18 days: 8/14	Early treatment (<18 days)	Late treatment (>18 days): more extensive deep stromal involvement (*p* = 0.022)
Chew et al. (2011) [76]	59/59	N/A	Initial VA (>0.40 vs. ≤0.40): OR: 4.3 (0.9–21.7) Time to diagnosis (≥21 vs. <21 days); OR: 1.6 (0.2–10.5) Steroid use before diagnosis: OR: 3.7 (0.4–15.7) Age (>50 vs. ≤50), yrs: OR: 2.3 (0.3–15.7) Diagnostic method (confirmed tissue diagnosis vs. clinical diagnosis) OR: 4.5 (0.8–25.1) Stromal involvement: (CRUDE OR:12.3 (2.4–62.7)) (adjusted: not estimable)
Bouheraoua et al. (2013) [27]	42/44	N/A	Higher rates of need for surgery: Time from symptom onset to diagnosis of >30 days: OR: 4.6 (0.3–83.5); *p* = 0.003 Initial visual acuity of ≤20/200: *p* = 0.01 Infiltrate size of >3 mm; *p* =0.03 Preperforating infiltrate; OR: 4.4; *p* < 0.001 Corneal neovascularization; OR: 7.0 (0.6–84.6); *p* < 0.001 Age ≥ 50 y: *p* = 0.001
Carnt et al. (2018) [13]	194/194	N/A	Presence of SIC (scleritis and/or a stromal ring infiltrate): Older age (>34 years): (OR: 2.36; 95% CI: 1.21–4.57; *p* = 0.011) Pre-diagnosis corticosteroid use: (OR: 2.56; 95% CI: 1.28–5.10; *p* = 0.008) Bad outcomes: Aged >34 years, OR: 2.52; 95% CI: 1.28–4.94; *p* = 0.007 Corticosteroids used before giving AAT (OR: 2.42; 95% CI: 1.17–5.03; *p* = 0.017) Symptom duration >37 days before AAT OR: 1.89; 95% CI: 0.91–3.90 Advanced disease stage (Stage 3, OR = 2.87, *p =* 0.010);
Chopra et al. (2020) [77]	157/157	BCVA < 0 (N = 75): No previous steroid therapy: OR (2.91; 1.00–8.10; *p* = 0.041) Clinical epithelial appearance (OR: 6.11; 2.32–16.07; *p* < 0.001) IVCM—ACD (number/mm^2^) (44.7 ± 40.1): OR:0.99; 0.98–0.99; *p* = 0.001 IVCM—morphologic features location (epithelium only): (OR: 14.22; 3.92–51.64; *p* < 0.001)	BCVA 0.18 to 0.78 (N = 55): Presence of corneal ring infiltrates and Severe stromal involvement. (OR: 3.30; 1.23–8.84; *p* = 0.02) IVCM—morphologic features location (Epithelium and stroma): (OR: 12.00; 3.22–44.74; *p* < 0.001) IVCM—Higher ACD (number/mm^2^) (48.2 ± 46.4): OR: 0.99; 0.98–0.99; *p* = 0.005
Bonini et al. (2021) [78]	35/40	Diagnosed early (<30 days) Prompt Acanthamoeba therapy (<30 days) (*p* < 0.01)	Severe corneal ulcer (stage III) had a significantly longer healing time (16.2 ± 3.7 months) (*p* < 0.05)
Rahimi et al. (2014) [37]	25/27	N/A	Deep stromal keratitis or a ring infiltrate: (OR), 28.0; 95% CI, 3.3–240.8, *p* = 0.001 Initial BCVA >0.3 logMar: OR, 8.6; 95% CI, 1.2–59.8, *p* = 0.003
Yamazoe et al. (2012) [79]	34/35 Group 1: final VA of ≤ 0.10 logMar (22 eyes) Group 2: >0.10 logMar (13 eyes)	N/A	Group 2 (>0.10 logMar) Initial BCVA > 0.50 logMAR: OR 25.5, 95% CI 3.4–186.7, *p* = 0.01 Diagnosis > 1 month: OR 1.03, 95% CI 1.00–1.06, *p* = 0.04 Presence of ring infiltrate: OR 33.6, 95% confidence interval (CI) 3.4–333.9, *p* = 0.01)
Arnalich-Montiel et al. (2014) [80]	17/17	N/A	Non-T4 genotype
Park et al. (1997) [81]	38/38	Early diagnosis (<1 month) Medical cure, *p* = 0.02 BCVA ≤ 0.48, *p* < 0.01	Late diagnosis (≥1 month)
Landeo et al. (2015) [82]	14/14	Early treatment (<30 days)	Late treatment (≥30 days) Higher duration of therapy (*p* = 0.0045) Poorer final BCVA (*p* = 0.0125)
Sun et al. (2006) [83]	20/20	N/A	N/A

Abreviations: N/A, Not Applicable; PHMB, polyhexamethylene biguanide; CCT, Corticosteroid; PKP, Penetrating Keratoplasty; AK, Acanthamoeba Keratitis; AAT, Anti-Amebic Therapy; BCVA, Best Corrected Visual Acuity; RGP, Rigid gas permeable lenses; PL, Perception of Light.

**Table 4:** Outcomes reported by included studies.

Article	No Patients/ Eyes	Good Oucome	Poor Outcome	Initial BCVA (logMAR Units) (Mean)	Final BCVA (logMAR Units) (Mean)	Improvement BCVA
Pang et al. (2024) [40]	109/109, results and outcomes were not available for 1 patient	22 (20%)	Corneal perforation, need for PKP, treatment >8 Months, BCVA ≥ 0.6 logMar, 86 (80%)	N/A	N/A	N/A
List et al. (2021) [41]	42/44	N/A	BCVA: ≥0.4 logMAR (54.5%), Need for KP (22.7%)	0.99 ± 0.73	0.56 ± 0.72	0.39 ± 0.68, *p* = 0.001
Megha et al. (2020) [38]	11/11, 1 lost in follow-up	Ulcers healed with vascularized corneal opacity (63.6%)	Need for KP (corneal perforation), 3 (27%)	≤0.60: 4 (40%) CF: 2 (20%) HM: 1 (10%)	0–1: 4 (40%) LP-HM: 3 (30%)	N/A
Jo et al. (2020) [42]	16/19	N/A	PHMB 0.02% + CHX 0.02%: Corneal toxicity in 2 /5 (40%)	0.78 ± 0.37	0.076 ± 0.07	PHMB: 0.89 ± 0.47 (*p* = 0.007) CHX: 0.59 ± 0.35 (*p* = 0.048) PHMB + CHX: 0.50 ± 0.22 (*p* = 0.042) Without statistically significant difference between groups.
Musayeva et al. (2020) [22]	28/28, 2 lost in follow-up	Medical cure (100%)	Need for KP (corneal scarring) (19.2%); Recurrence (repeated occurrence of symptoms and/or clinical signs of AK during or within 3 months after cessation of therapy); Stinging or burning sensation of the eye after application of the drops in 5 of 26 patients (19.2%)	1.02 ± 0.913 logMAR Stage I: 0.650 ± 0.354 Stage II: 0.843 ± 0.667 Stage III: 1.33 ± 0.974	0.504 ± 0.859 Stage I: 0.100 ± 0.141 Stage II: 0.286 ± 0.513 Stage III: 0.890 ± 1.07	*p* = 0.0004 Stage II: *p* = 0.028 Stage III: *p* = 0.1139
Papa et al. (2020) [43]	227/227	Medical cure:138 (60.79%) No statistical difference among AAT (*p* = 0.528)	Need to switch therapy: Diamidine monotherapy: 88% (22/25) PHMB + Diamidine: 24.6% (28/114) PHMB monotherapy: 48% (24/50) Others 52.6% (20/38) VA ≥ 0.6 logMar and/or need for surgery—112/227 (49.3%) (*p* > 0.155)	N/A	N/A	Severe vision loss ≥1.30 logMar: PHMB monotherapy: 11/50 (22%) PHMB + diamidine: 31/114 (27.19%) Diamidine monotherapy: 4/25 (16%) Others: 10/38 (26.32)
Lin et al. (2018) [44]	22/24	Cure: 20 (83.3%)	Need for PK: 4 (16.7%)	<0.18: 2 (8.3%) 0.18–1: 14 (58.3%) >1: 8 (33.3%)	>20/30: 17 (70.8%) 20/30–20/200: 4 (16.7%) <20/200: 3 (12.5%)	N/A
McKelvie et al. (2018) [45]	52/58	N/A	4 (with delayed diagnosis): 3 OPK; 1 TPK with posterior enucleation (perforated cornea)	N/A	N/A	Improvement: 74% Unchanged: 9% Worsened: 7%
Lim et al. (2008) [18]	55/56, only 51 eyes completed the study	N/A	Treatment success: PHMB 0.02%: 18 (78.3%) CHX 0.02%: 24 (85.7%) (*p =* 0.49)	Need for KP: PHMB 3/23 (13%) CHX 2/28 (7%) (*p* = 0.65) Corneal scarring (*p* = 0.29)	N/A	N/A
Wouters et al. (2022) [24]	109/109	N/A	Emergency corneal grafting: No CCT: 7/53 (13%); CCT: 20/56 (36%) (*p* = 0.0078) >1 surgery: No CCT: 9/53 (17%); CCT:22/56 (36%) (*p* = <0.0001) Diagnostic delay: No CCT: 23 ± 39 (range 7–303) days; CCT:62 ± 62 (range 0–295) days (*p* = <0.001) Higher disease severity stage with CCT use (*p* < 0.001)	N/A	<0.6 LogMar: No CCT:44(83%); CCT:35(63%) ≥0.6 LogMar: No CCT:7/53 (13%); CCT:17/56 (30%); (*p* = 0.03) Adjusted OR: CCT prior to AAT = 4.3 (95%CI (1.7–11.0)) (*p* = 0.002)	N/A
Thulasi et al. (2021) [23]	15/15	Clinical cure: 14 (93%)	GI disturbance: 7/15 (47%) Elevated liver functions: 2/15 (13%) Inflammatory response: 11/15 (73.3%) (10 improving with CCT) Surgery needed: 5/15 (33%) (1 enucleation (epithelial down growth)) Recurrence of AK and need more cycles of miltefosine: 6/15 (40%)	N/A	<0.6 logMar: 9 (60%) 1.4 logMar: 1(6.7%) HM: 2(13%) LP: 1(6.7%) No LP: 1 (6.7%)	N/A
Rahimi et al. (2015) [26]	31/31	Improvement in signs and symptoms: 26 (83.9%)	Need for adding another anti-AK agent: 4 (12.9%) Corneal scar: 8 (25.8%) Required OPK: 3 (9.7%)	<0.5: 22 (71%) ≥0.5: 9 (29.0%)	≥0.80: 22 (71%) <0.80: 9 (29%)	Better: 29/31 (93.5%) Same: 1/31 (3.22) Worse: 1/31 (3.22)
Robaei et al. (2014) [25]	174/174	N/A	Symptom duration (days) (Median (IQR): CCT: 37 (23–72.5); Not-CCT: 14 (7–28); *p* < 0.001 Scleritis, n (%): CCT: 44 (50.57%); Not-CCT: 16 (18.39%); *p* < 0.001 KP, n (%): CCT: 37 (42.53%); Not-CCT: 8 (9.20%); *p* < 0.001 Stage 3 AK: CCT: 31.8% vs. Not CCT: 15.5%, *p* = 0.037	N/A	N/A	CCT group: (Final Visual Acuity ≥0.6 logMar or Corneal Perforation or Need for Keratoplasty) OR: 3.90 (1.78–8.55); *p* = 0.001
Bagga et al. (2021) [33]	23/23, only 18 patients completed the study	Complete resolution: BG (40%); VZ (50%) Ulcer size BG group: 5.7 (5.3–6.5) to 1 mm (IQR, 0–4.3 mm) (*p* = 0.02) VZ group: 4.5 (1.8–5.1) to 0.7 mm (IQR, 0–1.6 mm) (*p* < 0.05)	Worsened keratitis: BG (30%); VZ (12.5%);	BG group: 1.79 (IQR, 1.48–2.78) VZ group: 1.60 (IQR, 1.00–2.78)	BG group: 1.10 (IQR, 0.48–1.79) VZ group: 0.80 (IQR, 0.48–1.30)	BG group: *p* = 0.02 VZ group: *p* = 0.18
Azuara-Blanco et al. (1997) [46]	10/10	N/A	N/A	From 0 to HM	≤0.3 logMar: 8/10 (80%) CF: 2 /10 (20%)	Improved in 100%
Duguid et al. (1997) [21]	105/111	N/A	Need for OKP: 10 (9%); Clinical relapses (tapering therapy): 19 (17%) Treatment toxicity (26.1%) (Propamidine—superficial punctate keratopathy) Glaucoma: 4 (3.6%) leading to 2 enucleations	From 0.30 to 2.30 logmar	≤0.30: 88(79.3%) ≥0.78: 18 (16.2%); included central scarring (52.2%), bacterial superinfection (30.4%), and recurrent disease (26.1%).	N/A
Hargrave et al. (1999) [34]	83/87, only 60 eyes had data analyzed for treatment efficacy and safety	Successful treatment without recurrence of infection after cessation of therapy 50 (83%)	PK: 17 (28%) Enucleation: 7/17 (41%) (due to incomplete eradication of infection prior to surgery) (*p* < 0.001). Treatment failure; exacerbations occurred during maintenance therapy, indicating quantitative inadequacy: 10 (17%) Propamidine-related toxicity included superficial punctate keratopathy 3/60 (3%): discontinued therapy due to severe burning on drop instillation.	N/A	N/A	Improved 83% of eyes evaluated
Carnt et al. (2016) [47]	196/196, only 129 included in the multivariable analysis	CCT use post-AAT and visual acuity ≥0.6, corneal perforation, or need for keratoplasty: OR: 1.08 (0.387–3.03); *p* = 0.881	CCT before diagnosis: 32 (25%) suboptimal outcomes	≥1	≤0.30 in 75%	N/A
Höllhumer et al. (2020) [48]	52/52	Voriconazole: Decreased the duration of AAT from the average of 12–9 months	Corneal scarring and vascularization, 32 (62%) Recurrence: 3 (5.8%) Need for surgery: 4 (7.7%) (1 TPK; 2 OPK; 1 DALK)	1.02 [0,4]	0.57 [−0.10 to 4.0] Stage 1: −0.03 ± 0.05 Stage 2: 0.50 ± 0.82 Stage 3: 1.32 ± 1.69, (*p* = 0.04).	27/52 (52%)
Caruso et al. (2020) [35]	29/29	18 (62%) ≤ 0.69 logMAR 15 (52%) ≤ 0.5 logMAR - Ocular inflammation improved at 2 weeks: 14 (48%)	10% (3/29) worsened to LP or lower Scarring, 24% (7/29) 1 (PKP or AMT)	1.76 ± 0.47.	0.77 ± 0.48 at 3 months	Significantly at 2 weeks, stable at 3 months (0.77 logMAR units)
Dart et al. (2024) [36]	135/135, 127 full-analysis subset; 134 in the safety analysis subset	Medical cure rate at 12 months: PHMB 0.02% + Propamidine, 86.6% PHMB 0.08%, 86.7% (*p* = 0.980)	Need for TPK PHMB 0.02+: 3/61 PHMB 0.08%: 5/66 Treatment failures: PHMB 0.02: 7 (11.5%) PHMB 0.08%: 10 (15.2%) Severe eye pain: 3 cases (2 in PHMB 0.02%, 1 in PHMB 0.08%)	N/A	Not statistically significant difference	N/A
Blaser et al. (2024) [49]	52/59, 46 were treated	Treatment success rate without KP: 97.8%	1: emergent PK due to conservative treatment failure	N/A	N/A	N/A
Tew et al. (2020) [50]	107/107, 10 AK eyes	Graft clarity 1 y post-op: 5 (50%) AK Cure: 9 (90%) Anatomical success rate: 9 (90%)	N/A	N/A	N/A	N/A
Chen et al. (2004) [51]	108/108, 15/15 with AK	Cure: 13 (86.7%)	Recurrence: 2(13.3%) Enucleation (scleral extension): 1 (6.67%)	0.54–1.30: 1(6.7%) 1.30: 14(93.3%)	0–0.48: 7 (63.6%) 0.54–1.30: 1 (9.1%) 1.30: 3 (27.3%)	Significant visual recovery observed in cases with clear grafts and no recurrence.
Laurik et al. (2019) [52]	23/23	Graft survival at 36 months: 78% (18/23) Early PK: 90% Late PK: 44% *p* = 0.167	Glaucoma *p* = 0.34 Cataract *p* = 0.16 Anterior synechiae *p* = 0.13	Early PK: 1.67 Late PK: 1.78 *p* = 0.418	Early PK: 0.32 Late PK: 1.28 *p* = 0.015	N/A
Liu et al. (2020) [53]	62/62	Medical cure: 52.4%	Post-op complications: TPK 82.4%; OPK 40%, *p* = 0.04 Glaucoma (*p* = 0.04): 58.8% TPK vs. 30% OPK; Recurrence: 2 eyes in TPK group	OPK: 1.57 ± 0.2 TPK: 1.79 ± 0.1 *p* = 0.52	OPK: 0.76 ± 0.2 TPK: 1.11 ± 0.2 *p* = 0.29	N/A
Kitzmann et al. (2009) [54]	30/31	Microbiological cure (>3 months without infection); Graft survival (TPK: 45.5% (1 y), 37.5% (10 y); OPK: 100% (1 y), 66.7% (10 y))	Repeated KP: 8 (in TPK group); Complications: corneal thinning, hypopyon (TPK), peripheral neovascularization (OPK)	No statistical difference between groups	Group OPK: 0.1 Group TKP: 0.30 Less likely to obtain visual acuity of 0.3 logMar or better (*p* 0.07);	N/A
Qi et al. (2024) [55]	51/51	Graft survival (DALK: 89.5%; PKP: 61.1%); (*p* = 0.046) Endothelial cell loss: DALK (1899 ± 125); PKP (1608 ± 231) (*p* = 0.032)	Autoimmune rejection of graft: PKP (20.8%) Recurrence (*p* = 1.000)	DALK: 1.95 ± 0.61 PKP: 2.93 ± 0.39 *p =* 0.039	At 1 Year: BB-DALK: 0.71 ± 0.59, PKP: 0.79 ± 0.66, *p* = 0.144 At 3 Years: BB-DALK: 0.71 ± 0.64, PKP: 0.93 ± 0.76, *p* = 0.010	N/A
Wang et al. (2023) [56]	13/13	Graft survival: 92.3%	Intraoperative Descemet membrane perforation: 1 (7.7%) Graft Rejection: 1 (7.7%) Recurrence: 1 (7.7%)	Ranged from HM to 1.7	0.35 ± 0.27	N/A
Zhang et al. (2023) [57]	58/59 with stage 3 AK requiring keratoplasty	Successful treatment: PKP 91.7%; LK 91.3%	Graft rejection: PKP: 8 (22.2%); LK: 0; *p* = 0.044 Graft epitelial deffects: PKP: 3(8.3%); LK: 0 Graft autolysis: PKP: 1(2.8%); LK:0 Secondary glaucoma: PKP: 3(8.3%); LK: 1(4.3%) Graft infection: PKP: 0; LK:1(4.3%) Recurrence: PKP: 6(16.7%); LK: 4(17.4%); *p =* 0.604	CF: PKP:16(44.4%); LK:18(78.3%); *p* = 0.016 HM: PKP:18(50.0%); LK: 4(17.4%) LP: PKP: 2(5.6%); LK:1(4.3%)	≤0.48: PKP: 14(38.9%); LK: 15(65.2%); *p =* 0.032 1–0.48: PKP: 12(33.3%); LK: 6(26.1%) >1: PKP:10(27.8%); LK:2(8.7%)	N/A
Roozbahani et al. (2019) [58]	63/63	N/A	TPK complications: graft failure (75%), cataract (50%), uncontrolled glaucoma requiring surgery (17%); recurrence, 8%	TPK: 2.55 ± 1.12; Medical: 0.82 ± 0.79	TPK: 1.83 ± 1.16; Medical: 0.43 ± 0.62	N/A
Sabatino et al. (2016) [59]	11/11	Recurrence: 0%; Endothelial cell density at 12 months: 2064 ± 443 cells/mm²; Graft rejection: 0%	Membrane rupture: 1 (9%)	Range HM to 0.5	0.84 ± 0.14	*p* < 0.1
Robaei et al. (2015) [60]	196/50	N/A	Need other eye surgery, n (%): TPK: 23 (88.5%); OPK: 14 (58.3%) Need multiple eye surgery, n (%): TKP: 7 (1 because of recurrence); OPK: 3 (none because of recurrence)	N/A	≤0.18: TPK: 54.2%, OPK: 26.5%; >1: TPK: 8.3%, OPK: 53.9%	TPK and Final VA >1 logMAR unit (OR: 12.78 (2.05 ± 79.72); *p =* 0.006)
Bagga et al. (2020) [61]	23/23, 10: Advanced AK (infiltrate ≥8 mm)	1 year graft survival: advanced AK: 32%; less severe: 91.6%	Graft Failure: Advanced AK (60%); less severe AK (15.4%); (OR: 8.25; *p* = 0.04) Complications in advanced AK: Descemet’s membrane detachment in 5/10 (50%); persistent epithelial defect in 3/10 (30%) Recurrence: Advanced AK: 2 cases (20%) Less severe AK: 1 case (7.7%)	Median: 2.78 (IQR, 1.79–3.0);	Median: 1.79 (IQR, 0.70–2.78)	Statistically significant improvement in BCVA for clear grafts; failure cases showed minimal improvement
Thebpatiphat et al. (2007) [62]	20/20	N/A	N/A	N/A	N/A	N/A
Butler et al. (2005) [63]	20/20	N/A	Complications: 16 (80%): corneal scarring (40%), recurrent disease (25%), cataract (15%), resistant disease requiring penetrating keratoplasty (10%), perforation (5%), bullous keratopathy (5%), and scleritis (5%) Need for PK: 7 (35%)	Ranged from 1 to CF	≤0.30: 75% ≥0.78: 5%	75% achieved 0.30 or better at last follow up Improvement in 90% (18 eyes) of cases; Worsened in two eyes (10%)
Chin et al. (2015) [64]	13/15	N/A	Treatment time: CCT group (162 ± 50.89 days) vs. non-CCT group (94 ± 45.24 days) (*p* = 0.012)	CCT group: 1.78 ± 0.60 Non CCT group: 0.62 ± 0.69	CCT group: 0.87 ± 1.23(−0.1 to 3) Non CCT group: −0.03 ± 0.49 (−1 to 0) *p* = 0.367	N/A
Alfonso-Muñoz et al. (2018) [65]	10/10 Superficial stroma: 3/10 (30%) Deep stroma (ring infiltrates or corneal abscesses): 7/10 (70%)	Medical cure: 3/3 (100% in superficial stroma group) CCT after 53 days of AAT improved symptoms	KP: 6/7 (86%) (in the deep stroma group) (perforation risk or ocular spreading)	HM: 4/10 (40%) CF: 1/10 (10%) 0.16–0.2: 3/10 (30%)	0.25–1	Visual acuity improved in all cases after treatment
Kaiserman et al. (2012) [66]	41/42	N/A	N/A	1.2 ± 0.6	0.87 ± 0.94	N/A
Ikeda et al. (2012) [67]	103/104, 28/29 with AK	N/A	Poor outcome defined: visual acuity ≥0.40 logMar at the last visit or a requirement of keratoplasty	N/A	N/A	N/A
Patel et al. (2010) [68]	25/25, 1 lost in follow up	N/A	N/A	N/A	Early diagnostic group (<21 days): 0.30 Late diagnostic group (>21 days): 0.90	N/A
Shimmura-Tomita et al. (2018) [69]	10/10	N/A	Severe group: 1 TPK; 3 with poor visual acuity (<0.2) at the last visit: 4 (40%) Mild Group: 6 good prognosis with final BCVA of 1.2: 6 (60%)	N/A	Mild Group: Full recovery (BCVA 1.2) in all cases	N/A
Zhang et al. (2014) [70]	29/29	N/A	Defined as the need for TPK	N/A	N/A	N/A
Elmer et al. (2008) [39]	65/72	Control: ≤0.1 logMAR	Case: ≥0.1 logMAR or PKP	N/A	N/A	N/A
Radford et al. (1998) [71]	243/259	N/A	N/A	N/A	≤0.30: Propamidine + PHMB: 99 (92%) Propamidine + Neomicine + PHMB: 21 (70%) Propamidina + Clorexidina:15 (100%)	N/A
Vilares-Morgado et al. (2024) [28]	46/51 BCVA final < 1 logMAR group: 31 (60.8%) BCVA final ≥ 1 logMAR group: 20 (39.2%)	BCVA final < 1 logMAR: 31 (60.8%)	BCVA final ≥ 1 logMAR: 20 (39.2%) 12 eyes (23.5%) undergoing evisceration/enucleation	Baseline BCVA: Group BCVA < 1: 0.8 ± 0.7 (n = 26 eyes); Group BCVA ≥1: 1.3 ± 0.9 (n = 12); *p* = 0.047	N/A	N/A
Randag et al. (2019) [72]	224/224	≤0.3 logMAR: 137 (61.2%)	Treatment failures (>0.30 logMAR and/or need for KP): 87 (38.8%)	N/A	>0.3: 56 (25%)	N/A
Bacon et al. (1993) [73]	72/77, 4 lost to follow up	≤0.3 logMAR units:58(79%) Recurrence caused graft failure in 9 of 21 inflamed cases	>0.48 logMAR units: 15 (21%)	N/A	N/A	Statistically significant improvement observed in 79% of cases, especially in early-diagnosed cases (*p* < 0.001)
G D’Aversa et al. (1995) [74]	12/14	Medical Cure: 11 (79%)	2 (15%) had BCVA CF or worse, including 1 loss due to bacterial superinfection.	≥1	≤0.40: 12 (85%) ≤1: 6(43%) 0.40 to 0.18: 6 (43%) CF or worse: 2 (14%)	N/A
Claerhout et al. (2004) [75]	14/14	N/A	Need for KP: Early group: 1/6 (17%) (for corneal necrosis) Late group:5/8 (62.5%): 2 TPK; 3 OPK	Worse in late group (*p* = 0.022)	Early group (<18 d): 5/6 (83%): ≤1 Late group (>18 days): 3 (37.5%): ≥1	N/A
Chew et al. (2011) [76]	59/59	≤0.1 logMAR: 45% ≤0.7 logMAR: 69%	PKP: 13 (22%) >0.7 logMAR: 16 (31%)	Ranged from 0 to 1.68 [mean: 0.68; SD = 0.54]	N/A	Improvement in BCVA in 69% of patients
Bouheraoua et al. (2013) [27]	42/44	Medical cure: 34(77%)	Need for surgery	1.24	Not surgical: 0.38: (n = 34) Surgical: 1.93: (n = 10) (*p* < 0.0001)	Improvement in 77%
Carnt et al. (2018) [13]	194/194	Better outcomes in early diagnosis and non-SIC cases: 101 (52%)	Final VA ≥ 0.60, and/or corneal perforation and/or KP and/or other ocular surgery, except biopsy, and/or duration of AAT ≥ 10.5 months: 93 (48%)	N/A	N/A	N/A
Chopra et al. (2020) [77]	157/157	N/A	N/A	N/A	N/A	N/A
Bonini et al. (2021) [78]	35/40	Diagnosed early (<30 days) had faster healing (7 months) and better visual outcomes	Diagnosed late (>30 days): prolonged healing (16.2 months for stage III) and higher surgery rates (41%).	N/A	N/A	Significant improvement in early-diagnosed cases; poor outcomes in advanced-stage cases despite treatment.
Rahimi et al. (2014) [37]	25/27	N/A	KP: 5 (18.5%)	≤0.10: 4 (15%) 0.50 to 0.10: 11 (40%) ≥0.50: 12 (45%)	≤0.10: 18 (66.7%) >0.10: 9 (33.3%)	77.8%: improved 14.8%: worsened
Yamazoe et al. (2012) [79]	34/35 Group 1: final VA of ≤ 0.10 logMar (22 eyes) Group 2: >0.10 logMar (13 eyes)	N/A	N/A	(Mean ±SD) Group 1: 0.47 ± 0.42 Group 2: 1.59 ± 0.79	Group 1: ≤0.10 Group 2: >0.10	N/A
Arnalich-Montiel et al. (2014) [80]	17/17	N/A	N/A	N/A	T4 genotype group: ≤0.10 Non-T4 genotype group: >0.10	T4 genotype: no significant improvement; severe vision loss in all cases Non-T4 genotype: all cases improved to functional vision
Park et al. (1997) [81]	38/38	Medical cure CCT, 14 (73.3%); *p* = 0.58 Non-CCT, 13 (76.5%) CCT before diagnosis, 7 (77.8%); *p* = 0.67	TPK Non-CCT, 4 (23.5%) CCT, 5 (26.3%), *p* = 0.26 CCT before diagnosis, 2 (22.2%), *p* = (0.21) TPK (n = 15): Graft survival 2 y Uninflamed, 100% Inflamed, 43% Recurrence, 4	N/A	≤0.48 Non-CCT vs.: CCT, *p* = 0.26 CCT before diagnosis, *p* = 0.30	N/A
Landeo et al. (2015) [82]	14/14	N/A	Higher duration of treatment in late treatment cases (10 months vs. 6 months, *p* = 0.0045)	Early treatment group: 0.30 to 0.70 Late treatment group: 0.48 to CF	Early treatment group: 0.30 Late treatment group: 1.30 *p* = 0.0125	N/A
Sun et al. (2006) [83]	20/20	N/A	N/A	N/A	≤0.70 in 7 eyes	N/A

Abreviations: N/A, Not Applicable; CHX, Chlorexidine; PHMB, polyhexamethylene biguanide; CCT, Corticosteroid; OPK, Optical Keratoplasty; PKP, Penetrating Keratoplasty; KP, Keratoplasty; AK, Acanthamoeba Keratitis; AAT, Anti-Amebic Therapy; BB-DALK, Big-Bubble Deep Anterior Lamelar Keratoplasty; BCVA, Best Corrected Visual Acuity; CF, Counting Fingers; HM, Hand Motion; LP, Light Perception.

### 3.3. Risk of Bias in Studies

Figure S1, Figure S2, Figure S3 and Figure S4 display the findings of the risk of bias assessment of the included studies. Through quality assessment, 70% of studies were classified as “Good/Low risk of bias”, 28% as “Fair/Moderate risk of bias”, and 2% as “Poor”.

In the evaluation of the observational studies using the NIH Quality Assessment Tool for Observational Cohort and Cross-Sectional Studies, criteria 6 (exposure assessment before the outcome), criteria 10 (exposure assessment conducted multiple times over time), and criteria 12 (blinding to exposure status) were deemed not applicable to all studies that have a retrospective design. None of the studies justified their sample size but given the rarity of the disease, all were considered appropriate. Six articles were rated as “Fair” [37,42,49,54,68,80] primarily because they did not adjust for confounding variables, while two prospective studies [37,80] did not assess exposure more than once over time, and did not mention any blinding. The study by Radford CF et al. (1998) [71] was rated as “Poor” because it also lacked information regarding the selection process.

Regarding randomized controlled trials, Lim et al. (2008) [18], was rated as “Some concerns in risk of bias” because of the randomization process, due to missing outcome data, measuring the outcome (because of subjective measurements), and in the selection of the reported results due to the lack of prospective trial registration.

In case series using the risk of bias assessment with NIH Quality Assessment Tool for Case Series Studies, six studies were rated as “Fair” because of the lack of well-described statistical methods (criteria 8) [34,45,56,63,64,73], twelve studies were rated as “Fair” because of a lack of comparability between the subjects, as baseline variety was not addressed (criteria 4), and tools to mitigate this bias such as adjustment for cofounders or multivariate analysis were not used. The study by Alfonso-Muñoz et al. (2018) [65] was rated as “Fair” for also lacking a clearly stated purpose/study question and information about the selection of cases (criteria 3).

Case–control studies [24,39] were classified as “Good” because they applied all criteria correctly, even though they did not report concurrent use of controls and blinding. Wouters KA et al. (2022) [24] did not report a justification for their sample size, but given the rarity of AK, 109 eyes is considered an appropriate sample size.

### 3.4. Medical Therapy

#### 3.4.1. Amebicides

Biguanides and diamidines were the main classes addressed throughout most studies [18,21,26,36,38,41,42,43,45,46,63,65].

Regarding monotherapy with biguanides, Rahimi et al. (2014) studied PHMB 0.02% monotherapy in 27 eyes, demonstrating its effectiveness in the treatment of AK, with improvement in visual acuity in 77.8% of eyes, with 66.7% presenting ≤ 0.10 logMAR units [37]. However, 14.8% worsened, and 33.3% of eyes showed > 0.10 logMAR units [37]. Five eyes (18.5%) required keratoplasty (three TPK and two lamellar keratoplasty (LK)) [37]. Additionally, Megha et al. (2020) [38], described 11 cases of AK. All were given PHMB 0.02% eye drops, gradually tapered according to the response to treatment, presenting a cure rate of 63.6% (7 out of 11 eyes), with complications such as corneal scarring and vascularization. A total of 27.3% progressed to corneal ulcer perforation and required TPK [38]. One patient experienced a persistent infection after keratoplasty, leading to phthisis bulbi. Only 36.3% of patients retained relatively good visual acuity (1 to 0 logMAR units). Others experienced significant visual impairment due to corneal scarring [38].

In 2015, Rahimi et al. did an interventional prospective case series with 31 eyes treated with CHX 0.02% as monotherapy, showing its effectiveness in AK and suggesting it could be a good choice for initiating treatment. Improvement in signs and symptoms was detected in 83.9% (23 eyes). Visual acuity improved in 93.5% of patients, with 71% achieving a final visual acuity of ≤0.097 logMAR units. Only three patients (9.7%) required OPK and corneal scarring was observed in eight patients (25.8%) [26].

A comparison of these two treatment options was conducted in two studies, in which monotherapy with either PHMB or CHX was effective for the treatment of AK, with no significant differences in the resulting outcomes or the serious adverse effects [18,42]. In a randomized controlled trial with 56 eyes, Lim et al. (2008) compared the efficacy of PHMB 0.02% monotherapy (23 eyes) and CHX 0.02% monotherapy (28 eyes), initiated hourly day and night for the first two days, then reduced to hourly by day for the next five days, and finally four times daily according to the response to treatment. The following outcomes in PHMB and CHX groups were not statistically different: the success rate (78.3% vs. 85.7%, *p =* 0.49), the need for TPK (13% vs. 7%; *p* = 0.65) and the improvement in visual acuity (56.5% vs. 71.4%; *p* = 0.54) [18]. BCVA worsened in four patients from the PHMB group (17.4%) and in three patients from the CHX group (10.7%) [18]. Similarly, a retrospective review of 19 AK eyes by Jo et al. (2020) reported significant improvement (*p* < 0.001) in visual acuity with either monotherapy with PHMB 0.02%, CHX 0.02% monotherapy and a combination of the two biguanides, with no significant differences found between them [42].

Regarding the combination of biguanides and diamidines, nine studies considered various regimens [21,27,36,41,43,45,46,63,65]. Medical cure, improvement in final BCVA, and signs and symptoms such as photophobia and pain were evaluated in all articles. Poor outcomes were also described but in a minority of cases, the majority being related to the need for surgery, corneal scarring, and recurrent disease.

Three studies retrieved conclusions regarding the association between PHMB 0.02% and propamidine isothionate 0.1%, gradually tapered according to the response to treatment [21,46,63]. For instance, Azuara Blanco et al. (1997), described a series of 10 eyes, with complete medical cure without recurrence after treatment completion and an improvement in visual acuity in levels ranging from hand motions to ≤0.30 logMAR units in 80% of eyes. Two eyes required OPK [46]. Duguid et al. (1997) described a series of 111 eyes, with 79.3% (88/111) of patients achieving BCVA ≤ 0.30 logMAR units and 16.2% ≥0.78 logMAR units because of corneal central scarring (52.2%), bacterial superinfection (30.4%), and recurrent disease (26.1%). Glaucoma was reported in four eyes leading to two enucleations [21]. Finally, Butler et al. (2005) showed an improvement in visual acuity in 90% of eyes, with complications in 80% due to a delay in diagnosis [63]. One study assessed the combination of PHMB 0.02% and hexamidine 0.1%, with a 77% (34/44 eyes) cure rate with medical treatment alone and better final BCVA (average of 0.38 logMAR units) [27]. In two studies, the biguanide and diamidine were not specified, but both reported an improvement in BCVA (74% of eyes in the study by McKelvie et al. [45], and an improvement of 0.39 ± 0.68 logMAR units (*p* = 0.001) in final BCVA, in the study by List et al. [41].

Two studies presented comparisons between monotherapy and combination therapy (biguanides and diamidines) [36,43]. Dart et al. (2024) [36], in a randomized controlled trial with 127 eyes, demonstrated that PHMB 0.08% monotherapy was noninferior to PHMB 0.02% and propamidine 0.1%, both applied every hour for the first 19 days and tapered to four times daily for the next months. Medical success rates were similar (>86%) and average BCVA was 1 logMAR unit in both groups [36]. A total of 13.4% experienced treatment failure and 6.3% required TPK [36]. No serious drug-related adverse events were reported [36]. Moreover, treatment with biguanides and diamidines, in monotherapy or combined, was compared by Papa et al. in 2020 [43]. In their study, there were no statistical differences regarding the medical cure (*p* = 0.528) or poor outcomes (*p* = 0.155) between groups, and PHMB 0.02% monotherapy showed higher rates of the need to switch therapy compared to PHMB and diamidine (48% versus 24.6%) [43].

Finally, the ocular toxicity of biguanides and diamidines was described in most studies as not severe or significant and consisted mainly of stinging or superficial punctate keratopathy [18,21,22,34]. Nonetheless, Lim et al. (2008) reported that eyes treated with PHMB tended to have a greater degree of scarring, while eyes treated with CHX tended to have milder scarring [18].

#### 3.4.2. Antifungals

Bagga et al. (2021) [33] investigated the role of voriconazole as a first-line treatment for AK, in a randomized controlled trial that included 23 eyes. In their study, topical voriconazole 0.1% (VZ group) was compared with the association of biguanides (PHMB 0.02% and CLX 0.02%) (BG group). Both treatment regimens demonstrated similar safety and effectiveness, though the authors reinforced the need for more studies with a larger sample size and longer follow-up periods. Corneal ulcer size decreased significantly in both groups (VZ group: from 4.5 mm to 0.7 mm (*p* < 0.05) versus BG group: from 5.7 mm to 1 mm (*p* = 0.02)), although the improvement in BCVA was only significant in the BG group (1.79 logMar units to 1.10 logMar units (*p* = 0.02) vs. 1.60 logMAR units to 0.80 logMAR units (*p* = 0.18) in the VZ group) [33].

Multiple studies used topical voriconazole as an adjunctive therapy. However, its effect was not evaluated individually, except in the study by Musayeva et al. (2020) [22] in which 28 eyes were treated with PHMB 0.02%, propamidine 0.1%, and supplementary voriconazole 1%, when clinically required. In this study, adjunctive therapy with voriconazole appeared to be effective, with resolution of the AK in all eyes and a significant improvement of BCVA (1.02 ± 0.913 versus 0.504 ± 0.859 logMAR units, *p* = 0.0004). TPK was required due to corneal scarring in 19.2% of eyes, and recurrence was reported in 3.85% of eyes but resolved after re-treatment. Toxicity occurred in 19.2% of eyes but consisted only of a stinging or burning sensation after application [22]. Furthermore, Höllhumer et al. (2020) reported a decrease in the duration of AAT from an average of 12 to 9 months in patients treated with adjunctive oral voriconazole [48].

Itraconazole is another antifungal formally used as an adjunctive AAT. Although many studies described its use, none reported specific outcomes or conclusions about this drug [27,43,58,65,73,76,83].

#### 3.4.3. Corticosteroids

Corticosteroids’ use prior to confirmed diagnosis or at the start of AAT has been associated with a delay in diagnosis [24,45,57,64,68], more severe disease at presentation [24,25,69], and worse clinical outcomes [13,24,25,66,72,73].

Park et al. (1997) showed that steroid use was not associated with higher treatment failures (*p* = 0.67) or poor visual outcomes (*p* = 0.30) [81]. Nonetheless, multiple studies demonstrate that corticosteroid use led to delayed diagnosis [24,45,57,64,68]. For instance, Wouters et al. (2022) conducted a case–control study with 109 eyes, in which there was a significantly higher diagnostic delay in eyes previously treated with corticosteroids (average of 62 ± 62 days) when compared to the nonsteroid group (23 ± 39 days; *p* < 0.001) [24]. Similarly, Patel et al. (2010) [68] reported an average diagnostic delay of more than 21 days in the eyes previously treated with corticosteroids, while McKelvie et al. (2018) [45] reported an average diagnostic delay of 47 days (ranging from 15 to 154 days), and Chin Joyce et al. (2015) [64] reported a diagnostic delay of over 30 days, although this was not statistically significant (*p* = 0.367).

Furthermore, eyes previously treated with corticosteroids presented a more severe disease at presentation, with more advanced AK stages [24,25,69]. These eyes also required longer AAT, as reported by Chin Joyce et al. [64]. In their study, the corticosteroid group required significantly longer treatment (162 ± 50.89 days), when compared to the non-corticosteroid group (94 ± 45.24 days; *p* = 0.012) [64].

Worse initial visual acuity and worse outcomes were also described in eyes previously treated with corticosteroids [13,24,25,66,72,73]. Wouters et al. (2022) reported a higher rate of initial BCVA ≥ 0.60 logMAR units in the steroid group (30% vs. 13%; *p* = 0.03) [24], while Kaiserman et al. (2012) [66] reported a worse visual prognosis (final BCVA of 1.05 vs. 0.39 logMAR units; *p* = 0.04). List et al. (2021) [41] described an OR:7.33 (95% CI (1.34, 40.21)), associating CCT with a higher risk of final BCVA ≥0.4 logMAR units or the need for KP, and Randag et al. (2019) [72] reported a higher risk of AK medical treatment failure (OR: 3.308, 95% CI 1.375–7.963). Misdiagnoses, particularly with herpetic keratitis, were more common in the corticosteroid group, as reported by Robaei et al. (73.3% vs. 32.2%; *p* < 0.001) in 2014 [25]. In their study, the corticosteroid group also experienced longer symptom duration (median of 37 (23–72.5) days vs. 14 (7–28) days; *p* < 0.001) and worse outcomes, such as scleritis (50.57% vs. 18.39%; *p* < 0.001) [25]. Carnt et al. (2018) described a higher risk of corneal perforation and the need for surgery when the eyes were treated with corticosteroids before AAT (OR: 2.42; *p* = 0.017), as well as a higher incidence of inflammatory complications (such as scleritis or stromal ring infiltrates) when corticosteroids were given pre-diagnosis (OR = 2.56; *p* = 0.008) [13]. Finally, Wouters et al. (2022) observed a four times increased risk of poorer outcomes in this group of patients, including emergency corneal grafting and the need for more than one surgery (OR: 4.2 (95%CI (1.7–11.0)); *p* = 0.002) [24].

The use of corticosteroids after the initiation of AAT was described in many studies, particularly to improve the management of pain, discomfort, and inflammatory complications of AK [22,23,26,27,47,65]. Carnt et al. (2016) found that the association between the use of corticosteroids after the start of AAT and worse visual outcomes was not statistically significant (OR: 1.08, 95%CI (0.39–3.03); *p* = 0.881) [47]. Their introduction was often delayed to prevent worse outcomes [26,27,47,65], and the time of initiation varies among studies but most of them recommend a median delay of two weeks [26,27,47]. This delay is important to allow AAT medication to work and reduce the risk of aggravating the infection. Regarding the choice of CCT and dosing, there is no consensus among studies; however, Carnt et al. (2016) [47] stated that for mild to moderate cases prednisolone 0.5% is often used four times daily, while more severe cases may require dexamethasone 0.1%. Zhang et al. (2023) [57] suggests using a low concentration steroid (0.1% fluormetholone eye drops) 2–3 weeks after surgery if no typical signs of recurrence were present, administered twice daily for 1 week, after which the frequency increased to 4 times daily. Additionally, Bacon et al. (1993) [73] stated using prednisolone 0.3% or dexamethasone 0.1% for eyes with specific indications such as uveitis, indolent ulcers, stromal lysis, or after keratoplasty, while systemic steroids were reserved almost exclusively for scleritis combined with topical steroids. The duration of therapy varied but was generally tapered once inflammation resolves. Carnt et al. (2016) [47] stated that AAT should be continued four times per day for four weeks after the withdrawal of CCT and once the eye is free of inflammation, treatment with CCT is then discontinued.

#### 3.4.4. Antiparasitics

Miltefosine has also been used as a salvage therapy in patients with refractory AK. It has been reported as an effective and well tolerated AAT in these cases, though some eyes may require increased corticosteroid therapy due to secondary severe ocular inflammation [23,28]. Thulasi et al. (2021) performed a retrospective multicenter case series of 15 eyes with refractory AK treated with oral miltefosine as salvage therapy. A clinical cure was described in fourteen cases (93.3%), but five required surgery (one enucleation), six (40%) suffered a recurrence and needed more cycles of miltefosine, and eleven (73.3%) experienced severe inflammation, ten of which were resolved with corticosteroids. Regarding toxicity, gastrointestinal effects were the most common and adverse effects [23]. Vilares-Morgado et al. (2024) reported the use of oral miltefosine in two cases, with poor visual outcomes (BCVA > 1 logMAR unit) [28].

#### 3.4.5. Other Therapies

Some studies mentioned the inclusion of antibiotherapy in their medical treatment regimens. For example, Neosporin^®^ (neomicin + polimyxin B + gramidicin) has been used in many studies [34,62,65,66,71,76]. In 1999, Hargrave et al. [34] published a prospective multicenter series which included 60 eyes with AK, all treated with propamidine isethionate 0.1% and neosporin^®^. This regimen resulted in an 83% cure rate, but at least 1 year of treatment was necessary. Neomycin, polymyxin, bacitracin, and other antibiotics were also mentioned in other studies, but none specifically investigated their effects.

A study by Caruso et al. (2020) [35] explored a solution containing CHX 0.02% and VE-TPGS. This combination was effective in most cases of their study, with significant improvement in BCVA (1.76 to 0.78 logMar units at 3 months) and anterior segment inflammation, with no active corneal inflammatory signs at 3 months. However, corneal scarring (65.51%), persistent stromal opacities (65.5%), and failures of treatment requiring additional anti-AK therapy (24.13%) were frequent in their study, representing major concerns for this therapy.

### 3.5. Surgical Therapy

#### 3.5.1. Therapeutic Epithelial Debridment (TED)

The studies conducted by Lin et al. (2018) [44] and Vilares-Morgado et al. (2024) [28] provided further insights regarding the treatment of AK, highlighting the effectiveness of TED using ethanol for 30 s beforehand in AK.

Lin et al. (2018) reviewed 24 cases where this therapy was applied [44]. Twenty cases (83.3%) were successful, not requiring any further surgical treatment. In contrast, the remaining four needed salvage TPK. Additionally, 80% of the cases demonstrated an improvement in BCVA of more than 0.2 logMAR units [44].

Similarly, this technique was associated with a significantly better visual outcome in the study by Vilares Morgado et al. (2024) (OR for BCVA <1 logMAR units: 19.02; 95%CI 3.27–110.57; *p =* 0.001) [28]. In their study, although TED was mostly performed in stages 1 and 2 of AK (63.6% of all procedures), even when performed in stage 3 AK the procedure led to better visual and morphological outcomes [28]. In fact, out of the eight eyes that presented with stage 3 AK and underwent TED, seven eyes presented a good final visual outcome (final BCVA < 1 logMAR units). Additionally, epithelial debridement significantly reduced disease severity, need for surgery, and recurrence rates [28].

Blaser et al. described a protocol that began with TED, followed by the use of propamidine 0.1% and PHMB 0.02% [49]. This protocol reported a 97.8% success rate, with only one out of fourty-six eyes (2%) requiring PK due to conservative treatment failure [49].

#### 3.5.2. Therapeutic or Tectonic Penetrating Keratoplasty (TPK)

In several studies, TPK was effective in eradicating AK refractory to medical treatment, with success rates ranging between 86.7% to 93.3% [50,51,56,57]. However, graft transparency 1 year post-operatively varied between 50% (5/10) [50] and 78.6% (11/14) [51], with smaller graft sizes (<8.5 mm) associated with better outcomes [50,51]. Furthermore, complications were common with corneal scarring, graft failure, cataracts, and glaucoma being described in some studies [51,58].

Final BCVA varied across studies but showed a reserved prognosis overall. Zhang et al. (2023) [57] showed that 94.4% of eyes had BCVA ≤ counting fingers, while Roozbahani et al. (2019) [58] observed an average of 2.55 ± 1.12 logMAR units after TPK, significantly worse than in those treated medically (vs. 0.82 ± 0.79 logMAR units; *p* < 0.01). Bacon et al. (1993) [73] stated that 79% of eyes post-TPK had ≤ 0.30 logMAR units. Lastly, Chen et al. (2004) [51] demonstrated 93.3% of eyes with final BCVA >1.30 logMAR units.

Early surgical intervention with TPK improved outcomes such as final BCVA, graft survival, and complications [51,52]. Laurik et al. (2019) compared early and late PK in a cohort of 23 eyes with treatment-resistant AK, demonstrating that eyes treated with TKP in the first 5.3 months within the onset of symptoms have a significantly better final BCVA compared to eyes that underwent TPK after 5.3 months (0.32 ± 0.18 logMAR units vs. 1.28 ± 0.89 logMAR units; *p* = 0.015) [52]. In their study, though success rates were reasonable, TPK led to ocular complications such as secondary ocular hypertension or glaucoma, anterior synechia, and cataracts [52]. Furthermore, graft failure and endothelial rejection were more frequent in the late TKP group, though this difference was not statistically significant [52]. Chen et al. (2004) stated that an early intervention with TPK should be made when medical treatment fails, adding that smaller graft sizes had better outcomes (<8,5 mm), reporting a cure rate of 86.7% [51].

Recurrence after TPK was a common problem mentioned in some studies [51,57,81]. For instance, in the study by Zhang et al. (2023) [57], AK recurrence varied from 16.7% (with PK) to 17.4% (with lamellar keratoplasty). The use of corticosteroids before AAT and hypopyon development were the main risk factors for AK recurrence [57]. Chen et al. (2004) described two cases of AK recurrence (out of fifteen AK cases, 13.3%), with one eye requiring enucleation due to scleral extension of the infection [51].

#### 3.5.3. Optical Penetrating Keratoplasty (OPK)

Four studies compared the rates of success, visual outcomes, complications, and graft survival rate between OPK and TPK [53,54,60,73]. OPK was performed after the resolution of active keratitis and delayed until the eye was not inflamed is associated with a better visual prognosis [53,54,60,73]. Graft survival was also significantly higher in OPK [54,73], as is final BCVA, with the majority of patients achieving a final BCVA of under 0.30 logMAR units [54,73]. Complications were more common in TPK than OPK, particularly glaucoma, which were more frequent in larger grafts, and dilated pupil/iris atrophy, as described by Liu et al. in [53] (2020). AK recurrence was more frequent in eyes that underwent TPK [53], often requiring multiple grafts (36% vs. 0% in the study by Kitzmann et al.) [54]. Robaei et al. (2015) stated that TPK had 12.78 more chances of final visual acuity > 1 logMAR units compared to OPK (*p* = 0.006) [60].

#### 3.5.4. Deep Anterior Lamellar Keratoplasty (DALK)

Studies suggest a higher graft survival and better visual outcomes with DALK in AK [55,56,59]. Qi et al. (2024) demonstrated that big-bubble DALK (BB-DALK) offered higher graft survival rates at three years post-operatively when compared to PK (89.5% versus 61.1%; *p* = 0.046) [55]. Similarly, Wang et al. (2023) [56], reported a 92.3% graft survival rate with elliptical DALK in severe cases of AK, contrary to Bagga et al. (2020) [61], who observed the opposite with lower survival rates in advanced AK (32% versus 91.6%). Both studies classified cases with a large infiltrate size (≥8 mm) as advanced AK. However, these studies differed in terms of the depth of corneal involvement in AK, because Bagga et al. included cases with deeper stromal involvement, while Wang et al. excluded full-thickness infections [56,61]. These differences highlight the impact of disease stage in AK treatment with DALK. Ultimately, Sarnicola et al. (2016) achieved 100% graft survival with early DALK, performed within 30 to 60 days of symptom onset [59].

In most studies, DALK showed a better visual outcome [55,56,59,61]. Qi et al. (2024) demonstrated a higher improvement in BCVA in eyes treated with DALK when compared with PK (final BCVA of 1.95 ± 0.61 logMAR units versus 2.93 ± 0.39 logMAR units; *p =* 0.039), as did Wang et al. (2023), who reported an improvement of BCVA from 1.7 logMAR units to 0.35 ± 0.27 logMAR units (*p* < 0.001) [55,56]. In the study by Sarnicola et al. (2016) [59], early DALK proved to be a good treatment option, with an improvement in BCVA from hand motion to 1.30 logMAR units initially, to a final BCVA of 0.076 ± 0.072 logMAR units (*p* < 0.01). Visual outcomes were less favorable in advanced AK cases treated with DALK, with Bagga et al. (2020) reporting minimal improvement in failed grafts [61]. Nonetheless, there was a statistically significant improvement in clear grafts, with overall BCVA improving from a median of 2.78 (IQR: 1.79–3.0) to 1.79 (IQR: 0.70–2.78) logMAR units [61].

Even though these studies showed a low rate of complications with DALK in early-stage AK [55,56,59], Bagga et al. reported a higher rate of graft failures in severe AK (OR: 8.25; *p* = 0.004), as well as complications, including a 50% rate of Descemet’s membrane detachment, and 60% graft failure [61]. In terms of AK recurrence, the overall rates across the studies were low, with Bagga et al. [61] reporting a 20% recurrence rate (two out of ten cases of severe AK), Qi et al. [55] reporting an 11.3% recurrence rate (three cases out of twenty-seven), and Wang et al. [56] describing one case of AK recurrence (7.7%).

### 3.6. Prognosis Factors

Several studies highlight key prognostic factors associated with worse outcomes.

Advanced disease at presentation, particularly deep stromal involvement or ring infiltrates (stage 3 AK), is a strong predictor of poor visual outcomes in AK [27,37,53,65,77,79], often leading to the need for PK [13,37,39,47,55,67,70,72,77,78,79] and a longer healing time [78]. The reported odds ratios for poor visual prognosis range from 2.87 to 33.6, though the definition of poor visual prognosis varies between studies [13,37,39,47,55,67,70,72,77,78,79].

Presence of keratic precipitates [69], epithelial defects [66], infiltrate size >3 mm, and corneal neovascularization [27] are associated with poor outcomes and the need for surgical treatment [27,66]. IVCM signs of late-stage disease, such as clusters, chains, high cyst density or deep location of cysts (≥250 μm), are independent predictors of poor outcomes, including a higher risk of requiring TPK [70,77].

Delayed diagnosis and treatment initiation in AK has also been consistently associated with poorer visual outcomes across studies [13,18,21,25,27,28,37,39,44,50,58,62,63,65,68,70,71,72,73,74,75,76,78,81,82]. Median delays of more than 18 to 21 days from symptom onset to AAT initiation were associated with higher rates of surgery [39,50,68,75,76], more advanced stages at presentation [62,75], and, consequently, worse visual outcomes [13,25,68,70,72,78]. Other studies demonstrated that delays exceeding one month were associated with a significant increase in recurrence rates, ocular complications, need for surgical interventions, duration of treatment, and graft failure [27,63,68,73,74,79,81,82]. In 1997 and 1998, Duguid et al. [21] and Radford et al. [71], reported that patients diagnosed early (within 14 to 18 days from symptom onset) have lower relapse rates, lower need for surgery, and better visual outcomes, while more recently, in 2024, Vilares-Morgado et al. [28] demonstrated that an early diagnosis (within 14 days after the symptom onset) plays a critical role in achieving better visual outcomes (BCVA <1 logMar units). Advanced disease presentation, marked by deep stromal infections, is more frequent in cases of delayed diagnosis, requiring longer treatment and often surgical interventions [65].

Regarding the delay in time from symptom onset to AAT, most studies indicate that delays of 21 days or less are associated with a more successful treatment, while delays of 25 to 37 days significantly increase the risk of corneal perforation and need for TPK [13,44,58]. One study reported that a delay of over 56 days was significantly associated with unsuccessful outcomes [18].

Initial BCVA has been reported as an important predictor of the course of the disease and of final BCVA in several AK studies [27,37,41,58,76,79]. Roozbahani et al. (2019) [58] and Bouheraoua et al. (2013) [27] described a significant association between the initial BCVA ≥1 logMAR units and need for TPK, while Chew et al. (2011) [76] reported that an initial BCVA >0.40 logMAR units was significantly associated with a higher risk of requiring TPK and presenting a final BCVA >0.1 logMAR units. Rahimi et al. (2014) [37] and Yamazoe et al. (2012) [79] noted poorer prognosis when initial BCVA was >0.3 and 0.50 logMAR units, respectively. Finally, both List et al. (2021) [41] and Kaiserman et al. (2012) [66] described significant correlations between initial and final BCVA.

As previously documented in section “3.4.3 Corticosteroids”, the use of corticosteroids before initiating AAT is often associated with worse outcomes in AK. Nonetheless, use of corticosteroids during AAT can improve pain and inflammatory complications of AK, especially when its introduction is delayed.

Another important prognostic factor is the age of the patient with AK, though the age for which the prognosis is significantly worse has not been unanimously reported [13,25,27,41,47,60,69,76]. In some studies, older age at presentation is associated with worse outcomes when the age of the patient is over 50 years [25,60,76]. Other articles have reported that even an age of over 33 to 37 years can be significantly associated with worse visual outcomes in AK [13,41,47,69], as well as an increased rate of corneal perforation, need for surgery, severe inflammatory complications [47], and a final BCVA ≥ 0.4 logMAR units [41]. Finally, further studies reported that an age of at least 60 [25] or 70 years [60] is significantly associated with a worse prognosis. Randag et al. (2019) also demonstrated that the odds of medical treatment failure increased per year of age, resulting in a 1.66 times higher risk of medical treatment failure with an age increase of ten years [72].

Regarding the *Acanthamoeba* genotype, Pang et al. (2024) [40] described a better AK prognosis in eyes with that are infected with the T4C genotype, as opposed to an infection with the T4D genotype. Arnalich-Montiel et al. (2014) [80] stated worse outcomes in non-T4 genotype cases.

## 4. Discussion

AK is an emerging concern in corneal infections. Although considered a rare condition, the number of cases is increasing globally [62]. This disease can lead to severe ocular complications, including blindness [21]. The lack of studies, particularly systematic reviews and clinical trials, addressing standardized management protocols emphasizes the significance of our research.

We conducted a systematic review that thoroughly analyzed and synthesized the evidence from 61 full-text articles on the therapeutic management and prognostic factors of this corneal disease. These studies provide valuable insights into medical and surgical treatments, potential complications, and prognostic factors that clinicians should be aware of.

Firstly, studies reported that TED and topical ethanol (20%) are effective initial treatment options for AK [28,44,49]. Research demonstrated an 83.3% success rate, along with improvement in visual acuity [44], a favorable prognosis (BCVA < 1 logMAR unit), and reductions in disease severity, the need for surgical intervention, and recurrence rates [28]. Blaser et al. described a treatment protocol initiated with epithelial debridement, followed by an association of propamidine 0.1% and PHMB 0.02%, stating great results with a success rate of 97.8% [49]. Furthermore, these authors recommended inpatient care for patients with a confirmed diagnosis or strong clinical suspicion, emphasizing the importance of close monitoring. For outpatients, regular follow-up visits were advised to maintain a high level of treatment adherence, which is crucial for successful management of the condition [49].

Building on this, we found that the association between biguanides and diamidines has been reported by studies as a successful first-line medical therapy, especially in early-stage AK. Articles showed improvement in final visual acuity, signs and symptoms, and in medical cure above 77% [21,27,41,45,46,63]. Nonetheless, poor outcomes were not excluded, and Duguid et al. described corneal scarring, bacterial superinfection, recurrent disease, and glaucoma as some complications to account for [21]. Toxicity was reported in a minority of cases, primarily involving mild adverse reactions. The most common effects included stinging and superficial punctate keratopathy, particularly associated with the use of biguanides and, to a greater extent, diamidines [18,21,22,34]. Regarding the posology of this combination therapy, most studies initiated treatment with a high-frequency dosing regimen, administering the medication hourly, both day and night, for the first 48 to 72 h [21,27,45,46,49,63,65]. This was followed by hourly administration during the day only for the next 72 to 120 h [21,27,49,65]. Subsequently, the dosage was gradually tapered based on the patient’s medical response [21,27,41,45,46,49,63,65]. Some studies also reported the continuation of therapy for a duration of 3 to 12 months [21,45,49,65].

For a simpler treatment approach, some studies described the use of biguanides as monotherapy [18,26,37,38]. When comparing biguanide monotherapy to the association of biguanides with diamidines, most studies did not find statistically differences [36,42,43,79]. However, the majority of articles that study a biguanide and diamidine combination report better outcomes [21,46,63], including more promising results and a lower prevalence of complications, than studies focused solely on biguanide monotherapy [26,37,38]. These studies’ population was similar, with average ages ranging between 20 and 33 years, and including eyes with AK stages from I to III [21,26,37,38,46,63]. Dart et al. (2024) argued that monotherapy with PHMB 0.08% was non-inferior to combination therapy with a biguanide and propamidine (PHMB 0.02% and propamidine 0.1%), achieving high medical cure rates (>86%) without significant drug-related adverse effects [36]. However, we believe that further research with larger and more comparable sample sizes is needed to assess the potential toxicity and adverse outcomes associated with higher concentrations of PHMB [36].

Voriconazole, an antifungal agent, has been shown to induce programmed cell death [84] in Acanthamoeba and is effective against both trophozoite and cyst forms in vitro [84,85]. Bagga et al. [33] suggested that topical voriconazole 1% was effective and comparable to biguanide association therapy; however, only biguanides demonstrated a significant improvement in visual acuity. The authors emphasized the need for further trials with larger sample sizes and longer follow-up periods, as their study included only 23 eyes [33]. Despite these limitations, voriconazole demonstrated favorable outcomes with minimal side effects when used as an adjunctive therapy in combination with PHMB 0.02% and propamidine isethionate 0.1% [22]. Voriconazole may also be administered orally, with one study reporting a reduction in the average duration of AAT [48]. We conclude that voriconazole requires more clinical trials to confirm its efficacy as a monotherapy and first-line treatment. However, as adjunctive therapy, topical voriconazole 1% is a promising option due to its lower systemic toxicity compared to the oral formulation [86]. Additionally, miltefosine has demonstrated good in vitro activity against certain species of Acanthamoeba [85]. Our findings indicated that oral miltefosine was successfully used in cases of treatment-resistant AK, including instances where voriconazole had been previously administered [23]. However, its use was associated with side effects, including inflammatory responses in almost all cases, leading to pain, discomfort, and reduced patient well-being. Gastrointestinal symptoms such as nausea and vomiting, along with abnormal liver function tests, were also reported [23]. Vilares-Morgado et al. [28] associated miltefosine with poor prognostic outcomes, although this may be attributable to the drug’s use in patients presenting with more severe disease. Further studies are needed to clarify its role and effectiveness in the management of AK.

Regarding adjunctive therapy, we recommend the use of topical voriconazole 1% as the initial option. If the AK proves refractory, oral miltefosine may be considered. While miltefosine has demonstrated good cure rates, it is associated with more systemic and ocular side effects, which should be carefully weighed in the decision-making process.

Neosporin^®^ (neomicin + polimyxin B + gramidicin) and especially neomycin are a widely used as an adjunctive antibacterial therapy alongside other anti-amoebic agents, with favorable outcomes reported [34,62,65,66,71,76]. Although neomycin is not cysticidal, it plays a role by reducing the trophozoite population and preventing bacterial superinfection, which serves as an energy source for Acanthamoeba species [12,87]. This mechanism can support overall treatment effectiveness by limiting factors that promote the parasite’s survival and replication [12,87].

Lastly, the use of corticosteroids in the management of AK has been a subject of significant controversy. Our review found that corticosteroid use prior to a confirmed diagnosis or the initiation of AAT is associated with worse clinical outcomes. By temporarily improving the initial clinical presentation, corticosteroids can mask the true severity of the infection, leading to diagnostic delays (averaging between 21 and 62 days) [24,45,57,64,68] and a higher rate of misdiagnosis, particularly as herpetic keratitis [25]. These delays in initiating appropriate treatment were linked to more severe disease presentations, including advanced disease stages [24,25,69], prolonged treatment durations, and increased complications such as scleritis, corneal perforation, and the need for surgical interventions [13]. Additionally, studies consistently reported poorer outcomes, including lower BCVA and a higher risk of treatment failure [13,24,25,66,72,73]. These findings justify caution when considering corticosteroid use before diagnosis is established.

On the other hand, corticosteroid therapy, when used as adjunctive treatment, was often associated with the improvement of inflammatory symptoms, helping to minimize adverse effects. This benefit was observed when corticosteroids were introduced at least two weeks after the initiation of AAT [22,23,26,27,47,65]. Based on these findings, we recommend avoiding corticosteroids in the management of AK, particularly before diagnosis and the commencement of AAT. However, corticosteroids may offer therapeutic benefits when used to control inflammation, especially if their administration is delayed until after the infection is under control. Regarding the choice and dosing of CCT, there is no unanimity among studies but it often depends on the severity of inflammation and clinical response.

DALK has demonstrated favorable outcomes, with success rates exceeding 85% [55,56,59], good BCVA, and low recurrence rates (<11%), even in advanced cases of AK with infiltrates ≥ 8 mm [56,61]. However, in cases with deep stromal involvement, the procedure showed lower graft survival rates (60%), a high incidence of Descemet’s membrane detachment (50%), and increased recurrence rates (20%) [61]. Based on these findings, we conclude that DALK is a viable option for managing AK but should be avoided when deep stromal involvement is present due to its reduced effectiveness and higher complication rates [61].

TPK is described as an effective salvage therapy for eradicating infections refractory to medical treatment [50,51,56,57], particularly when smaller graft sizes are used, which are associated with better prognostic outcomes [50,51]. Nonetheless, TPK is also linked to a higher prevalence of complications, including corneal scarring, graft failure, secondary ocular hypertension (glaucoma), cataracts, recurrence, and an increased risk of enucleation [51,52,58]. However, TPK in essentially performed in the medically unresponsive and advanced stages of AK which are associated with poor prognosis and could explain the worse outcomes [58,60]. Findings suggest that TPK can be considered as a salvage therapy, and early intervention should be performed when treatment fails [51,52]. Smaller graft sizes should be considered [51,52].

OPK, when performed after the resolution of active keratitis and delayed until the eye shows no signs of inflammation, demonstrated superior outcomes compared to TPK [53,54,60,73]. These included better visual prognosis, higher graft survival rates, and lower recurrence rates [53,54,60,73]. Based on these findings, we conclude that OPK is best suited for rehabilitation purposes, providing improved long-term outcomes for patients who have recovered from active AK.

The choice of the surgical technique for managing Acanthamoeba keratitis (AK) depends on the stage of the disease and the patient’s clinical condition. Therapeutic penetrating keratoplasty (TPK) is the most effective option for refractory cases with significant stromal involvement, whereas deep anterior lamellar keratoplasty (DALK) is beneficial in early-stage AK, particularly when there is no deep stromal involvement. Optical penetrating keratoplasty (OPK) is generally indicated after the resolution of the active infection, primarily for visual rehabilitation.

Although TPK has high success rates in eradicating the infection, outcomes may be less favorable, especially with larger grafts. OPK offers better graft survival but requires careful timing to minimize complications. DALK provides superior graft survival and visual recovery in cases without endothelial or deep stromal involvement; however, its use is limited in advanced cases due to lower graft survival rates.

Complications are more frequent with TPK, including graft failure, cataracts, glaucoma, and high recurrence rates. OPK generally has fewer complications, but glaucoma remains a concern, particularly in larger grafts. Although DALK has a lower complication rate in early-stage cases, it still carries risks in advanced cases, including graft failure and Descemet’s membrane detachment.

To improve outcomes in the management of AK, it is essential to understand the factors that influence prognosis. Although there is no unanimous consensus on the exact age threshold, older age, particularly over 50 years, has been identified by multiple studies as a poor prognostic factor [13,25,27,41,47,60,69,76], likely due to worse BCVA at presentation and weaker immune responses [25,60,76]. Some studies also report ages over 33 to 37 years old [13,41,47,69] and 60 [25] or 70 [60] years old to be associated with a worse prognosis. Additionally, poor initial BCVA [27,37,41,58,76,79], T4C genotype, when compared to T4D [40], and non-T4 genotype [80] were described as predictors of poorer outcomes. However, it is important to state that while the two previously mentioned studies suggest a potential role of genotyping as a prognostic factor, their sample sizes are relatively small, limiting the reproducibility of these findings. Larger studies are necessary to assess the generalizability of these statements. While these factors are beyond the control of medical management, other prognostic determinants can be addressed, including delayed diagnosis [13,18,21,25,27,28,37,39,44,50,58,62,63,65,68,70,71,72,73,74,75,76,78], and advanced disease stage at presentation [13,27,37,39,47,53,55,65,67,70,72,77,78,79], as well as the use of corticosteroids, particularly before diagnosis [3,13,24,25,45,64,66,68,72,73]. Furthermore, while evidence is not comprehensively gathered, we acknowledged that pre-existing ocular conditions may influence and complicate treatment. There are reports of cases of AK infections following surgical procedures; however, the evidence remains unaggregated [88]. Studies indicate that reducing diagnostic delays can significantly impact prognosis [39,50,68,75,76]. A median delay of 14 to 21 days has been associated with improved outcomes and may positively influence other prognostic parameters [21,28,71]. A prompt diagnosis and an early initiation of appropriate treatment are thus crucial in optimizing patient recovery and reducing complications.

Given the significant side effects associated with available therapies, it is crucial to continue exploring more efficient and less harmful options for managing AK. Numerous in vitro studies are investigating innovative approaches to combat AK. Promising new therapies include dihydropyridines [89], statins [84,90], advanced corneal crosslinking (CXL) [91], riboflavin/rose bengal-mediated PACK-CXL [92], and carbonic anhydrase inhibitors such as ethoxzolamide and dorzolamide [93].

Additionally, new therapeutic targets are under investigation, including the cellulose biosynthesis pathway [94], cyclic AMP phosphodiesterase RegA [95], and elements of Acanthamoeba’s complex redox system [96]. These areas of research hold significant potential for developing novel treatment strategies. Encouraging further investigations and clinical trials is essential to improve the management and outcomes of this challenging condition.

Lastly, several limitations must be acknowledged in this review. Firstly, the majority of the included studies were retrospective, with only three randomized controlled trials, which may introduce substantial bias and prevent any definitive causal analysis. Although we systematically reviewed the available literature, there were significant variations in participant characteristics, such as the type and severity of AK, as well as differences in treatment protocols across the various centers where the studies were conducted. Additionally, the outcomes measured, particularly BCVA, were not consistently evaluated using the same scale across studies.

These factors highlight the heterogeneity of study populations and methodologies, which hindered the feasibility of performing a meta-analysis. Consequently, the findings regarding correlations between treatment efficacy, complications, and prognostic factors should be interpreted with caution. Further standardized, high-quality studies are necessary to provide more robust evidence.

This review recommends initiating treatment for AK with TED and 20% ethanol. Following this, therapy with PHMB 0.02% and propamidine 0.1% should be initiated, in combination with neomycin or Neosporin^®^. If the condition proves resistant to this regimen, topical voriconazole 1% may be added, followed by oral miltefosine if necessary. For surgical management, DALK is suggested if there is no deep stromal involvement. In cases where DALK fails or is not feasible, TPK is recommended as a salvage option. Finally, once inflammation and active infection are resolved, OPK can be performed for visual rehabilitation. A higher age, delayed diagnosis, poor BCVA, and AK stage at presentation are important prognosis factors that should be acknowledged.

## Data Availability

All data generated and analyzed during this study are included in this published article.

## Supplementary Materials

The following supporting information can be downloaded at: https://www.mdpi.com/article/10.3390/jcm14072528/s1, Figure S1. Cochrane Risk of bias assessment for randomized controlled trials. (a) Traffic-light plot; (b) Summary plot. Figure S2. NIH quality assessment for case–control studies. (a) Traffic-light plot; (b) Summary plot. Figure S3. NIH quality assessment for case series studies. (a) Traffic-light plot; (b) Summary plot. Figure S4. NIH quality assessment for observational cohort and cross-sectional studies. (a) Traffic-light plot; (b) Summary plot. Table S1. The search query for each database. Table S2. Excluded articles in full text screening.

## Abbreviations

The following abbreviations are used in this manuscript:

AK	Acanthamoeba keratitis
BCVA	Best corrected visual acuity
ICVM	In vivo confocal microscopy
AAT	Anti-amoebic therapy
PHMB	Polyhexamethylene biguanide
CHX	Chlorexidine
CCT	Corticosteroids
TED	Therapeutic epithelial debridement
TPK	Therapeutic penetrating keratoplasty
OPK	Optical Penetrating Keratoplasty
DALK	Deep Anterior Lamellar Keratoplasty
