# Supplementary material for: Acanthamoeba Keratitis Management and Prognostic Factors: A Systematic Review
Source: J Clin Med. 2025 Apr 7;14(7):2528. doi: 10.3390/jcm14072528 (PMC11989993; doi:10.3390/jcm14072528)
Supplement: Supplementary file 1 [file jcm-14-02528-s001.zip › Table S1.pdf]

Table S1. The search query for each database.

**MEDLINE®**	(("acanthamoeba"[MeSH Terms] OR "acanthamoeba keratitis"[MeSH Terms] OR "amoeba"[MeSH Terms] OR ("acanthamoeba keratitis"[MeSH Terms] OR ("acanthamoeba"[All Fields] AND "keratitis"[All Fields]) OR "acanthamoeba keratitis"[All Fields]) OR (("amebae"[All Fields] OR "amoeba"[All Fields] OR "amoeba"[MeSH Terms] OR "ameba"[All Fields] OR "amebas"[All Fields] OR "amoebas"[All Fields] OR "amoebae"[All Fields]) AND ("keratic"[All Fields] OR "keratitis"[MeSH Terms] OR "keratitis"[All Fields] OR "keratitides"[All Fields])) OR (("amebae"[All Fields] OR "amoeba"[All Fields] OR "amoeba"[MeSH Terms] OR "ameba"[All Fields] OR "amebas"[All Fields] OR "amoebas"[All Fields] OR "amoebae"[All Fields]) AND ("cornea"[MeSH Terms] OR "cornea"[All Fields] OR "corneal"[All Fields]) AND ("infect"[All Fields] OR "infectability"[All Fields] OR "infectable"[All Fields] OR "infectant"[All Fields] OR "infectants"[All Fields] OR "infected"[All Fields] OR "infecteds"[All Fields] OR "infectibility"[All Fields] OR "infectible"[All Fields] OR "infecting"[All Fields] OR "infection s"[All Fields] OR "infections"[MeSH Terms] OR "infections"[All Fields] OR "infection"[All Fields] OR "infective"[All Fields] OR "infectiveness"[All Fields] OR "infectives"[All Fields] OR "infectivities"[All Fields] OR "infects"[All Fields] OR "pathogenicity"[MeSH Subheading] OR "pathogenicity"[All Fields] OR "infectivity"[All Fields])) OR (("parasitation"[All Fields] OR "parasites"[All Fields] OR "parasited"[All Fields] OR "parasites"[MeSH Terms] OR "parasites"[All Fields] OR "parasite"[All Fields] OR "parasitical"[All Fields] OR "parasitically"[All Fields] OR "parasitics"[All Fields] OR "parasiting"[All Fields] OR "parasitism"[All Fields] OR "parasitisms"[All Fields] OR "parasitization"[All Fields] OR "parasitize"[All Fields] OR "parasitized"[All Fields] OR "parasitizes"[All Fields] OR "parasitizing"[All Fields] OR "parasitology"[MeSH Terms] OR "parasitology"[All Fields] OR "parasitic"[All Fields] OR "parasitology"[MeSH Subheading]) AND ("keratic"[All Fields] OR "keratitis"[MeSH Terms] OR "keratitis"[All Fields] OR "keratitides"[All Fields]))) AND ("treatment"[Title/Abstract] OR "surgery"[Title/Abstract] OR "follow-up"[Title/Abstract] OR "complications"[Title/Abstract] OR "prognosis"[Title/Abstract] OR "management"[Title/Abstract] OR "therapy"[Title/Abstract])) NOT ("case reports"[Publication Type] OR "animal"[Title/Abstract] OR "rat"[Title/Abstract] OR "mice"[Title/Abstract] OR "case report"[Title/Abstract])
**SCOPUS®**	TITLE-ABS-KEY(("acanthamoeba keratitis" OR "acanthamoeba" OR "amoeba keratitis" OR (("amebae" OR "amoeba" OR "ameba" OR "amebas" OR "amoebas" OR "amoebae") AND ("keratitis" OR "keratic")) OR (("amebae" OR "amoeba" OR "ameba" OR "amebas" OR "amoebas" OR "amoebae") AND ("cornea" OR "corneal") AND ("infectability" OR "infectable" OR "infectant" OR "infected" OR "infection" OR "infections" OR "infective" OR "pathogenicity" OR "infectivity")) OR (("parasitation" OR "parasite" OR "parasites" OR "parasitic" OR "parasitism" OR "parasitized" OR "parasitizing" OR "parasitology") AND ("keratitis" OR "keratic"))) AND ("treatment" OR "surgery" OR "follow-up" OR "complications" OR "prognosis" OR "management" OR "therapy")) AND NOT ("case reports" OR "animal" OR "rat" OR "mice" OR "case report")
**Web of Science®**	TS=("acanthamoeba keratitis" OR "acanthamoeba" OR "amoeba keratitis" OR (("amebae" OR "amoeba" OR "ameba" OR "amebas" OR "amoebas" OR "amoebae") AND ("keratitis" OR "keratic")) OR (("amebae" OR "amoeba" OR "ameba" OR "amebas" OR "amoebas" OR "amoebae") AND ("cornea" OR "corneal") AND ("infectability" OR "infectable" OR "infectant" OR "infected" OR "infection" OR "infections" OR "infective" OR "pathogenicity" OR "infectivity")) OR (("parasitation" OR "parasite" OR "parasites" OR

	"parasitic" OR "parasitism" OR "parasitized" OR "parasitizing" OR "parasitology") AND ("keratitis" OR "keratic"))) AND TS=("treatment" OR "surgery" OR "follow-up" OR "complications" OR "prognosis" OR "management" OR "therapy") NOT TS=("case reports" OR "animal" OR "rat" OR "mice" OR "case report")
