# Supplementary material for: Acanthamoeba Keratitis Management and Prognostic Factors: A Systematic Review
Source: J Clin Med. 2025 Apr 7;14(7):2528. doi: 10.3390/jcm14072528 (PMC11989993; doi:10.3390/jcm14072528)
Supplement: Supplementary file 1 [file jcm-14-02528-s001.zip › Table S2.pdf]

Table S2. Excluded articles in full text screening.

Reason for exclusion	Article	Title	Journal
**Results were not presented for AK infection individually / Not isolated AK**	Sepulveda-Beltran *et al* (2022)	Rose Bengal Photodynamic Antimicrobial Therapy: A Review of the Intermediate-Term Clinical and Surgical Outcomes	American Journal of Ophthalmology
	Naranjo *et al* (2019)	Rose Bengal Photodynamic Antimicrobial Therapy for Patients With Progressive Infectious Keratitis: A Pilot Clinical Study	American Journal of Ophthalmology
	Pan *et al* (2016)	Corneal infection in Shandong peninsula of China: a 10-year retrospective study on 578 cases	International Journal of Ophthalmology
	Otri *et al* (2013)	Profile of sight-threatening infectious keratitis: a prospective study	Acta ophthalmologica
	Chidambaram *et al* (2018)	Epidemiology, risk factors, and clinical outcomes in severe microbial keratitis in South India	Ophthalmic epidemiology
**Wrong outcome**	Por *et al* (2009)	Acanthamoeba keratitis associated with contact lens wear in Singapore	American journal of ophthalmology
	Santos *et al* (2018)	Acanthamoeba keratitis in Porto Alegre (southern Brazil): 28 cases and risk factors	Parasitology research
	Iovieno *et al* (2014)	Acanthamoeba sclerokeratitis: epidemiology, clinical features, and treatment outcomes.	Ophthalmology
**Review**	Zazzo *et al* (2022)	Therapeutic Corneal Transplantation in Acanthamoeba Keratitis: Penetrating Versus Lamellar Keratoplasty.	Cornea
**Doesn't include 10 eyes of AK**	Anshu *et al* (2009)	Outcomes of therapeutic deep lamellar keratoplasty and penetrating keratoplasty	Ophthalmology

		for advanced infectious keratitis: a comparative study	
	Zhao *et al* (2010)	Genotyping of Acanthamoeba isolates and clinical characteristics of patients with Acanthamoeba keratitis in China.	Journal of medical microbiology
**Without full text**	Parija *et al* (2001)	Acanthamoeba keratitis in Pondicherry	The Journal of communicable diseases
	Raj *et al* (2015)	Role of topical chlorhexidine in treatment of acanthamoeba keratitis	Biomedical and Pharmacology Journal
	Wang *et al* (1997)	Clinical features and outcome of Acanthamoeba keratitis	Journal of the Formosan Medical Association
**Non-English language**	Kandori *et al* (2007)	Clinical management and prognosis of Acanthamoeba keratitis: A retrospective study of 11 cases at the Department of Ophthalmology of Osaka University Medical School	Folia Ophthalmologica Japonica
	Zhang *et al* (2008)	Clinical synthetic diagnosis and management of acanthamoeba keratitis	Ophthalmology in China
	Jin *et al* (2000)	Investigations on the diagnosis and treatment of acanthamoeba keratitis	Chinese Ophthalmic Research

Abreviations: AK, Acanthamoeba Keratitis.
