# Supplementary figures and images for: Acanthamoeba Keratitis Management and Prognostic Factors: A Systematic Review
Source: J Clin Med. 2025 Apr 7;14(7):2528. doi: 10.3390/jcm14072528 (PMC11989993; doi:10.3390/jcm14072528)

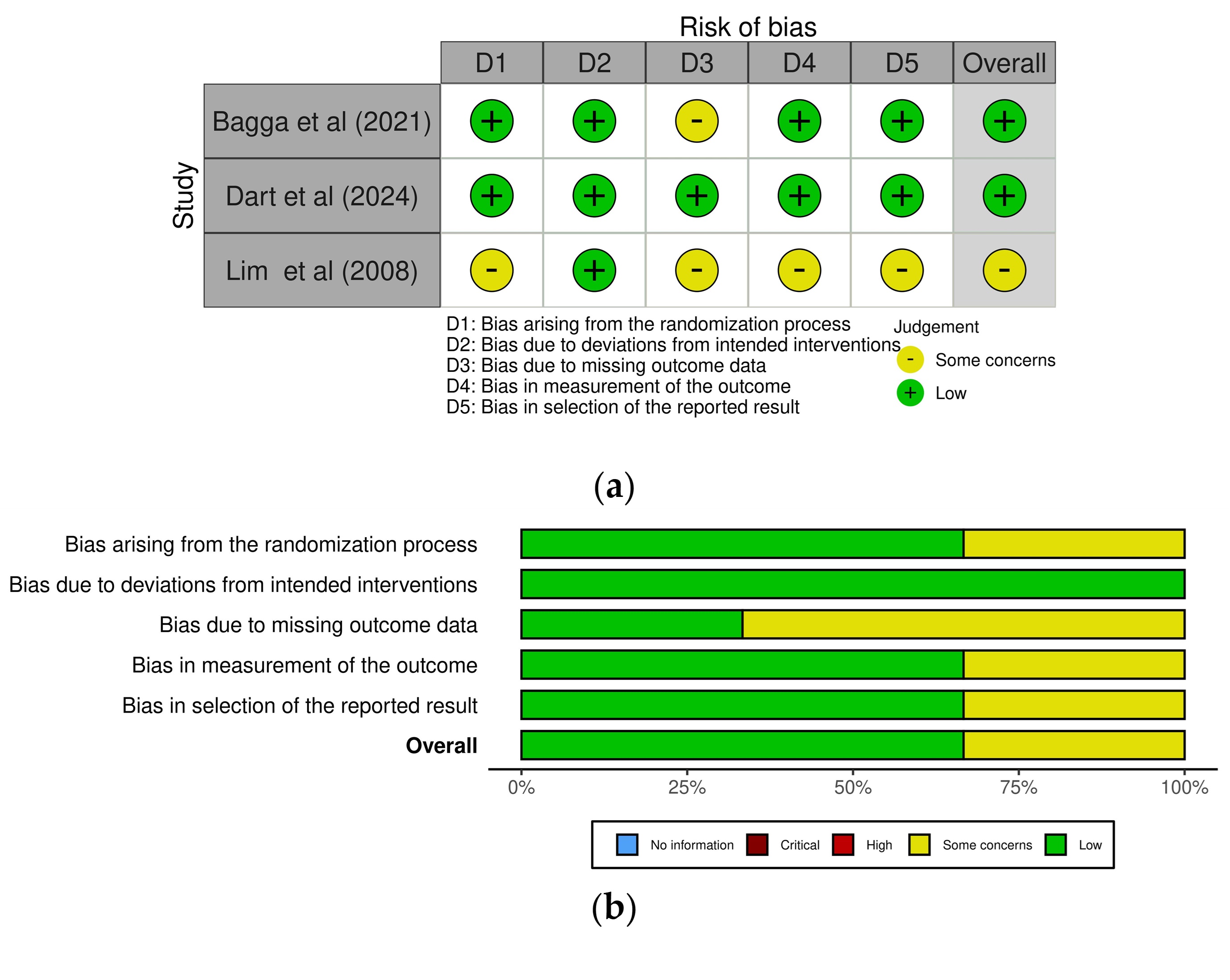

Supplement: Supplementary file 1 [file jcm-14-02528-s001.zip › Figure S1.jpg]

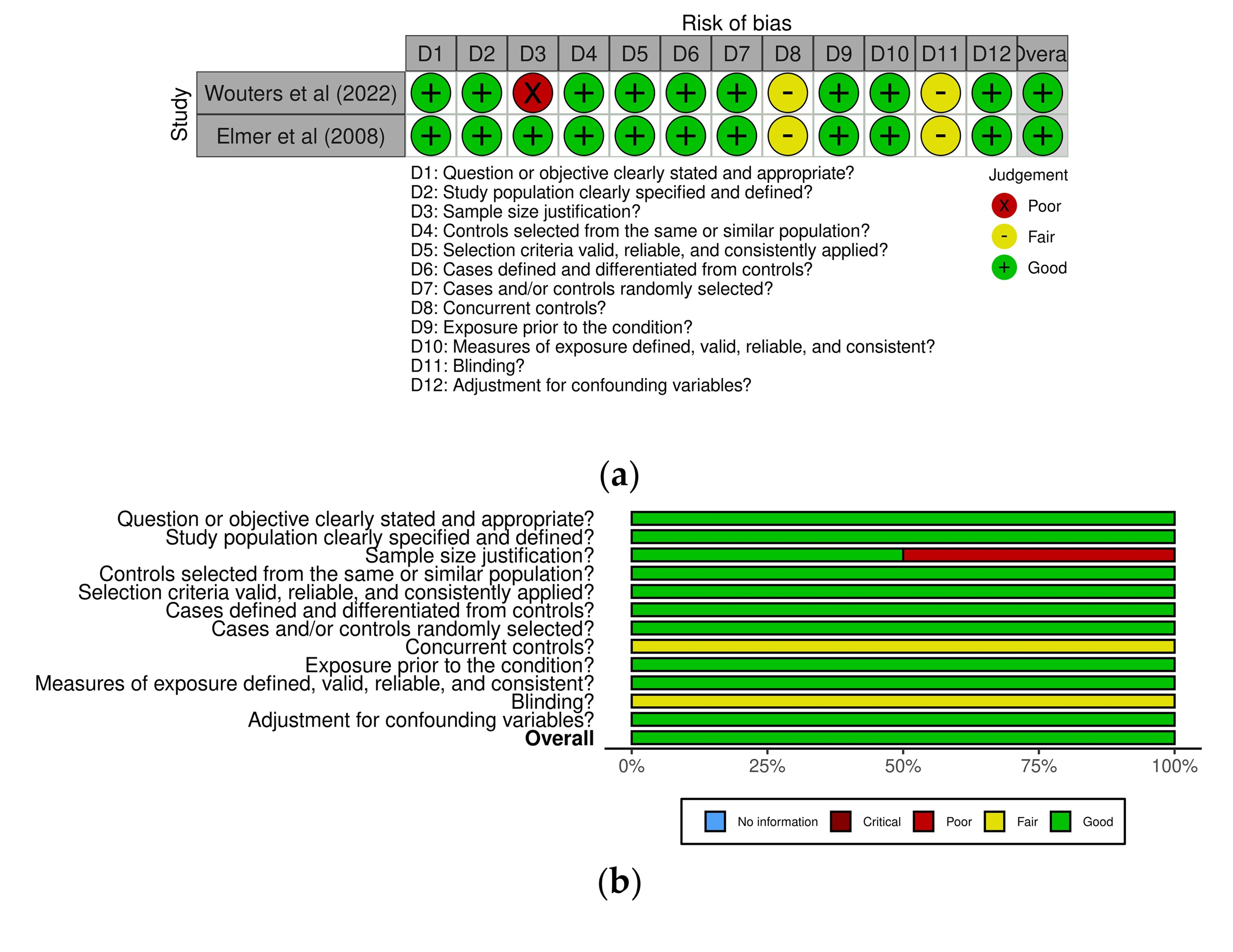

Supplement: Supplementary file 1 [file jcm-14-02528-s001.zip › Figure S2.jpg]

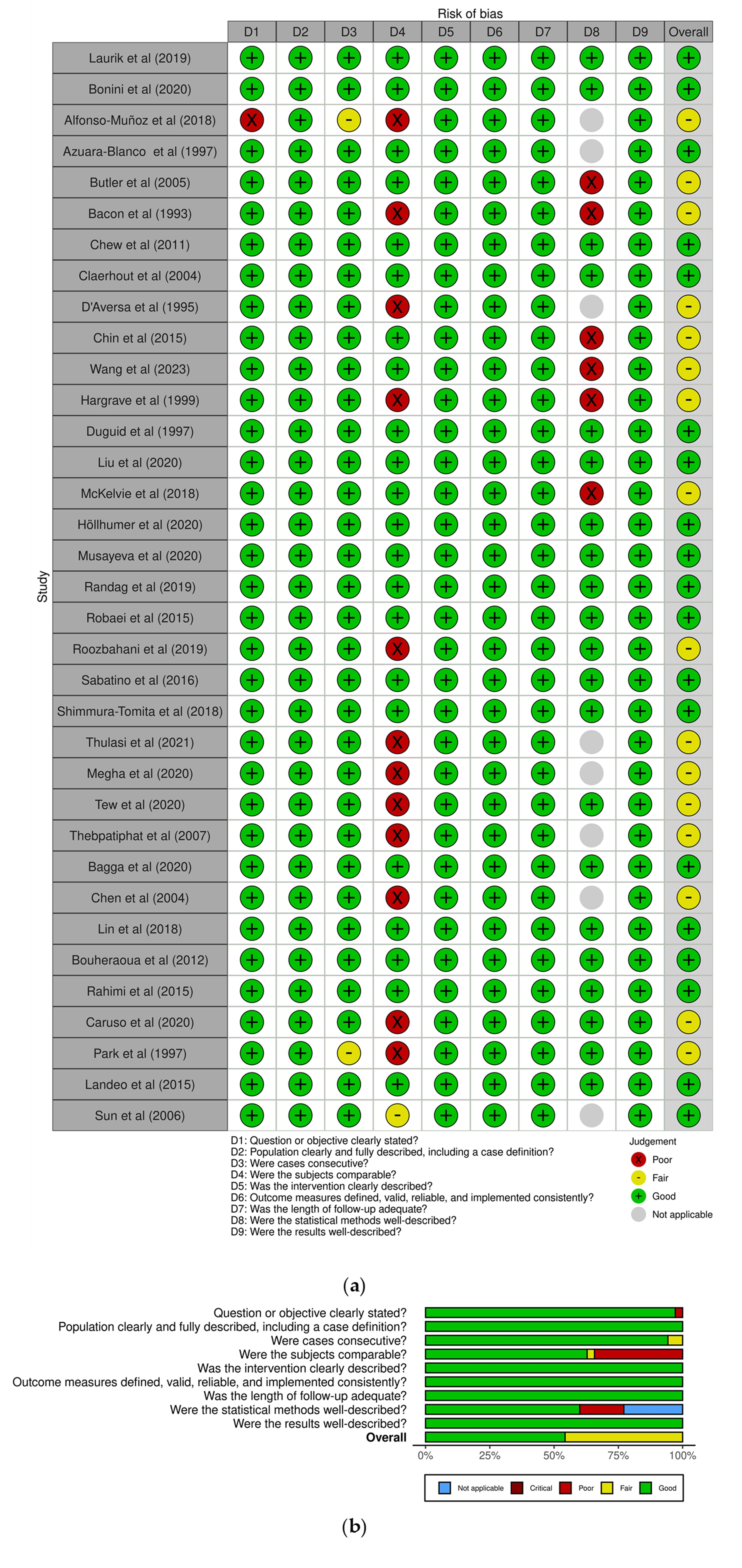

Supplement: Supplementary file 1 [file jcm-14-02528-s001.zip › Figure S3.jpg]

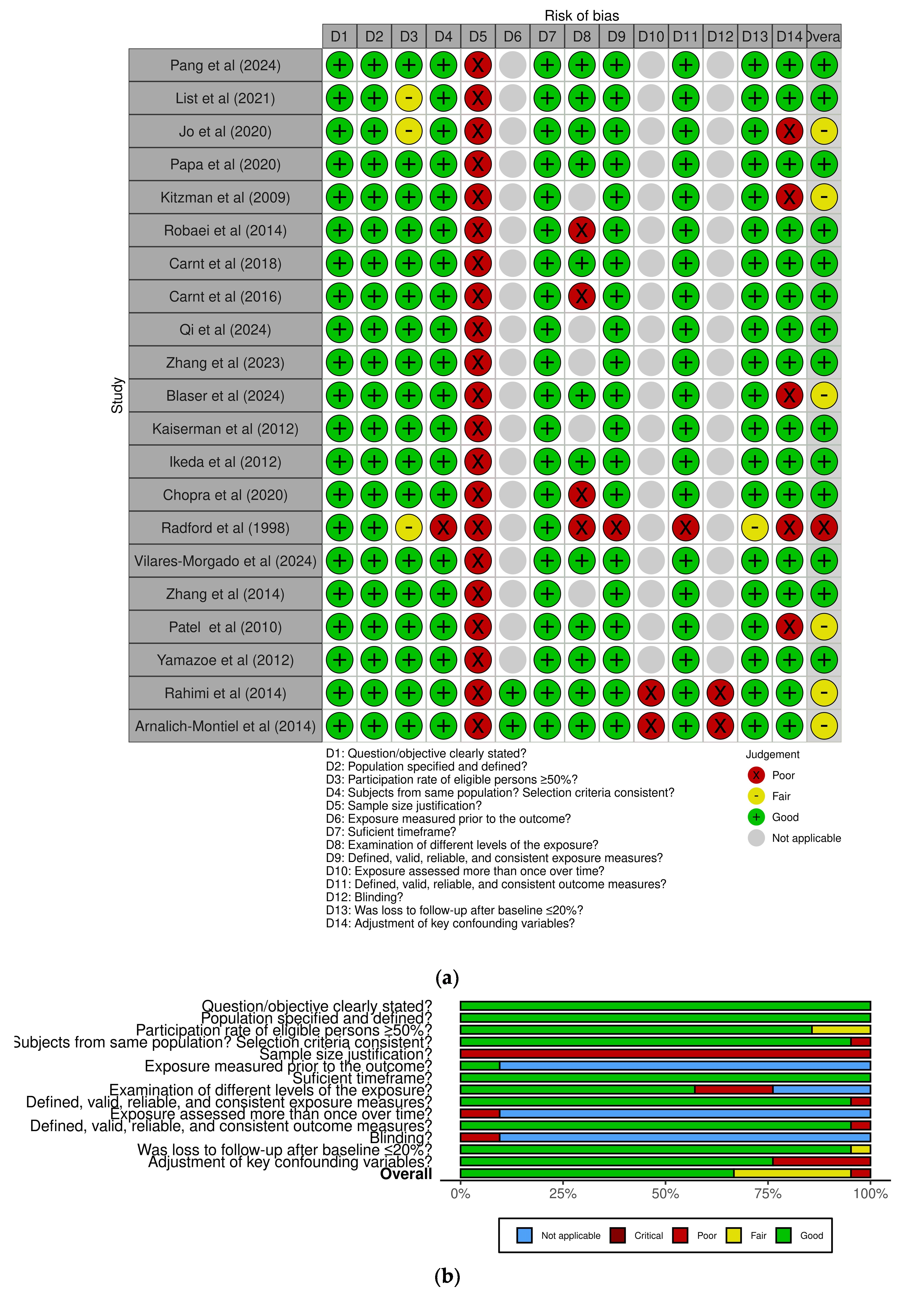

Supplement: Supplementary file 1 [file jcm-14-02528-s001.zip › Figure S4.jpg]
